# Pulmonary immune responses to *Mycobacterium tuberculosis* in exposed individuals

**DOI:** 10.1371/journal.pone.0187882

**Published:** 2017-11-10

**Authors:** Christian Herzmann, Martin Ernst, Christoph Lange, Steffen Stenger, Stefan H. E. Kaufmann, Norbert Reiling, Tom Schaberg, Lize van der Merwe, Jeroen Maertzdorf, for the Tb or not Tb consortium

**Affiliations:** 1 Center for Clinical Studies, Research Center Borstel, Borstel, Germany; 2 Division of Clinical Infectious Diseases, Research Center Borstel, Borstel, Germany; 3 German Center for Infection Research (DZIF), Clinical Tuberculosis Unit, Borstel, Germany; 4 International Health / Infectious Diseases, University of Lübeck, Lübeck, Germany; 5 Department of Medicine, Karolinska Institute, Stockholm, Sweden; 6 Institute for Medical Microbiology and Hygiene, University Hospital Ulm, Ulm, Germany; 7 Department of Immunology, Max Planck Institute for Infection Biology, Berlin, Germany; 8 Division of Microbial Interface Biology, Research Center Borstel, Borstel, Germany; 9 Center of Pneumology, Agaplesion Deaconess Hospital Rotenburg, Rotenburg, Germany; 10 LizeStats Consulting, Frankraal, Overstrand, Western Cape, South Africa; Institut de Pharmacologie et de Biologie Structurale, FRANCE

## Abstract

**Background:**

Blood based Interferon-(IFN)-γ release assays (IGRAs) have a poor predictive value for the development of tuberculosis. This study aimed to investigate the correlation between IGRAs and pulmonary immune responses in tuberculosis contacts in Germany.

**Methods:**

IGRAs were performed on bronchoalveolar lavage (BAL) cells and peripheral blood from close healthy contacts of patients with culturally confirmed tuberculosis. Cellular BAL composition was determined by flow cytometry. BAL cells were co-cultured with three strains of *Mycobacterium tuberculosis* (*Mtb*) and *Mtb* derived antigens including Purified Protein Derivative (PPD), 6 kD Early Secretory Antigenic Target (ESAT-6) and 10 kD Culture Filtrate Protein (CFP-10). Levels of 29 cytokines and chemokines were analyzed in the supernatants by multiplex assay. Associations and effects were examined using linear mixed-effects models.

**Results:**

There were wide variations of inter-individual cytokine levels in BAL cell culture supernatants. Mycobacterial infection and stimulation with PPD showed a clear induction of several macrophage and lymphocyte associated cytokines, reflecting activation of these cell types. No robust correlation between cytokine patterns and blood IGRA status of the donor was observed, except for slightly higher Interleukin-2 (IL-2) responses in BAL cells from IGRA-positive donors upon mycobacterial infection compared to cells from IGRA-negative donors. Stronger correlations were observed when cytokine patterns were stratified according to BAL IGRA status. BAL cells from donors with BAL IGRA-positive responses produced significantly more IFN-γ and IL-2 upon PPD stimulation and mycobacterial infection than cells from BAL IGRA-negative individuals. Correlations between BAL composition and basal cytokine release from unstimulated cells were suggestive of pre-activated lymphocytes but impaired macrophage activity in BAL IGRA-positive donors, in contrast to BAL IGRA-negative donors.

**Conclusions:**

*In vitro* BAL cell cytokine responses to *M*. *tuberculosis* antigens or infection do not reflect blood IGRA status but do correlate with stronger cellular responses in BAL IGRA-positive donors. The cytokine patterns observed suggest a pre-activated state of lymphocytes and suppressed macrophage responsiveness in BAL cells from BAL IGRA-positive individuals.

## Background

About one quarter of the world’s population is estimated to be infected with *Mycobacterium tuberculosis* [1]. Although the bacterium is transmitted via aerosol inhalation, the estimates for infection rates are based on non-respiratory assays. Both the tuberculin skin test (TST) and the blood based Interferon-γ release assay (IGRA) measure a systemic host immune response that is driven by T lymphocytes primed to *M*. *tuberculosis* antigens. The value of these two assays is controversial for several reasons. First, even after documented exposure to patients with contagious pulmonary tuberculosis, less than half of the contact persons develop a positive systemic immune response [2,3]. Second, individuals that are latently infected—as defined by a positive test in the absence of radiological or clinical signs suggestive of tuberculosis—rarely develop tuberculosis after exposure [4,5]. Third, TST and IGRAs may produce conflicting results in approximately 20% of the tested persons [6]. Fourth, it remains unknown whether a positive test is driven by persisting viable bacteria within the host or a lasting immune response to dead bacteria and mycobacterial antigens or based on memory responses in absence of nominal antigen. Evidence is accumulating that systemic immune phenomena only partially reflect pulmonary processes that determine a successful immune response against *M*. *tuberculosis* [7–14].

In order to better understand pulmonary immune responses after exposure to *M*. *tuberculosis*, we established an *ex vivo* cell culture based model that integrates *in vitro* antigen stimulation and mycobacterial infection of fresh human bronchoalveolar lavage (BAL) cells. The aim of this study was to identify local immune profiles that were associated with LTBI in order to (a) gather a detailed understanding of the steps that lead to an extrapulmonary immune response and (b) to augment the prediction of a systemic immune response in exposed individuals.

## Methods

This observational, multicentre, prospective study was carried out by the research consortium on “Pulmonary Tuberculosis—Host and Pathogen Determinants of Resistance and Disease Progression- (TB or not TB)”, funded by the German Ministry of Education and Research (BMBF, reference 01KI0784). The funder provided support in the form of salaries for one author [CH], but did not have any additional role in the study design, data collection and analysis, decision to publish, or preparation of the manuscript. The specific roles of these authors are articulated in the ‘author contributions’ section.”

The study protocol was approved by the ethics committee (EC) of the University of Luebeck, Germany (reference 07–125), and was adopted by other ethics committees covering all 18 participating centres (EC of the medical faculty of the University of Goettingen; EC of the Medical Council of Hessen, Frankfurt /Main; EC of the Medical Council Hamburg; EC of the Medical Council Lower Saxony, Hannover; EC of the Medical Faculty Carl Gustav Carus, Technical University of Dresden; EC of the Medical Council Berlin; EC of the Medical Council Bavaria, Munich; EC of the Medical Faculty, Friedrich-Alexander-University Erlangen-Nuremberg; EC of the Medical Faculty of the University of Regensburg; EC of the University of Witten/ Herdecke). A description of the cohort was recently published [15]. A study physician obtained oral and written informed consent from each individual before enrolment.

Household contacts (HHCs) without clinical and radiological evidence of active tuberculosis were recruited at three urban municipal healthcare centres (*i*.*e*., Frankfurt, Hamburg, Hannover). They were suitable for enrolment if (a) they were asymptomatic with no signs of tuberculosis on chest X-ray; (b) were exposed >8 hours in total to patients with detectable acid-fast bacilli (AFB) in the sputum and >40 hours in AFB negative, but culture-confirmed pulmonary; (c) their last unprotected exposure was at least 8 weeks prior to IGRA testing.

Adult healthcare workers (HCWs) with (a) ongoing professional contacts to patients with acid-fast bacilli sputum smear-positive tuberculosis; (b) a cumulative professional exposure of two or more years; and (c) no clinical/radiological signs or symptoms of active tuberculosis were recruited at 18 German pulmonary medicine centres (listed in the appendix).

Clinical and demographic data were captured on an *ad hoc* standardised questionnaire. IGRAs were performed on peripheral blood by the QuantiFERON Gold In-Tube^®^ (QFT; Cellestis Qiagen, Hilden, Germany). IGRA status in BAL samples were performed using the T-Spot.TB^®^ assay (Elispot; Oxford Immunotec, Oxford, UK) as published before [16–18]. According to the manufacturer’s instructions, the cut-off for a positive read out was set at 0.35 IU/ml IFN-γ for the QuantiFERON Gold In-Tube^®^. For the T-Spot.TB^®^ assay, the cut-off was set at ≥6 spots according to the package insert. Persons with known HIV infection or a significant pulmonary disease were excluded.

All enrolled healthy contacts were offered a bronchoscopy with bronchoalveolar lavage (BAL) unless bronchoscopy was contraindicated for medical reasons (e.g., poor venous access, medical conditions associated with an increased risk). If the person agreed, flexible bronchoscopy was performed according to current German guidelines with intravenous and local anaesthesia at the physician's discretion [19]. The bronchoscope was wedged into a subsegmental bronchus of the middle lobe. BAL was performed with a total volume of 200-250mL sterile normal saline. BAL fluid samples from a total of 25 blood IGRA-positive and 25 IGRA-negative donors were included in this study.

Data presented in this manuscript are from BAL samples processed on a single experimental site (Research Center Borstel) only.

Single-cell suspensions from BAL fluid were obtained by passing the BAL fluid through a stainless-steel sieve (Teesieb Profi Plus, WMF, Geislingen, Germany) with a mesh aperture of 0.5 mm. T-Spot.TB^®^ IGRA was performed according to the manufacturer’s instructions, with the exception that 200,000 mononuclear cells (instead of 250,000 mononuclear cells) were plated per well for improved visibility of the spots [20]. Elispot assays for BAL and blood were done as published before [21]. The assay was considered positive if ≥6spot-forming cells (SFCs) were counted in the ESAT-6 or the CFP-10 well after subtraction of the number of SFCs in the negative control well and if the total number of SFCs in the ESAT-6 or CFP-10 well was at least twice the number of SFCs in the negative control well.

Composition of the cells in the BAL fluid was analyzed by flow cytometry (FACSCalibur; Becton Dickinson, Heidelberg,Germany), using monoclonal antibodies against CD3 (Invitrogen, Carlsbad, CA, USA, Catalogue No. MHCD0305 and clone X35, Beckman-Coulter, Krefeld, Germany), CD4 (Invitrogen, No. MHCD0401), CD16 (Invitrogen, No. MHCD1604), CD25 (Invitrogen, No. MHCD2505), CD40 (Invitrogen, No. CD4005), CD45RO (Invitrogen, No. MHCD45RO04), CD62L (Invitrogen, No. MHCD62L05), CD66b (clone 80H3, Beckman-Coulter, Krefeld, Germany), CD127(IL-7R)α (R&D Systems, now Bio Techne, Minneapolis, MN, USA, No. FAB 306P), HLA-DR (BD Pharmingen Inc, San Diego, CA, USA, No. 347402), TLR-4 (NatuTec ebioscience, Frankfurt/Main, Germany, No. 13-9917-80). Viable cells were gated according to the FSC/SSC characteristics and the frequency of fluorescent cells within the complete gate was determined.

The major BAL subsets, namely monocytes/macrophages, lymphocytes, and granulocytes, could be determined by their characteristic patterns in the forward scatter (linear scaling) versus side scatter (logarithmic scaling) dotplots. More than 0.2 million BAL cells were acquired per vial out of 7 vials per BAL cell preparation stained with different combinations of fluorochrome-labelled monoclonal antibodies. The fractions of lymphocytes, monocytes, granulocytes, and macrophages in BAL cells were determined due to their respective scatter patterns which were based on a total of more than 1.4 million of BAL cells.

BAL lymphocyte subsets were defined as CD3^+^CD4^+^CD45R0^+^ and CD3^+^CD8^+^CD45R0^+^ cells for memory T lymphocytes, CD4^+^CD25^+^CD127^-^ cells for regulatory-T-lymphocytes, CD3^+^CD16^+^ cells for natural killer T cells, CD3^+^CD62L^+^ for naïve T cells, BAL neutrophilic granulocyes as CD66b^++^CD16^++^ and eosinophilic granulocytes as CD66b^++^CD16^+/-^cells within the granulocyte gate, while medium to strong expression of MHC class II could be demonstrated on macrophages and monocytes within the respective scatter-plot gates. In contrast to BAL lymphocytes, granulocytes, and monocytes, the surface antigen expression densities on BAL macrophages were assessed only qualitatively by immunofluorescence means with flow cytometry because of the strong autofluorescence of BAL macrophages seen in most cases.

For antigen stimulation assays in 96-well plates, 200,000 BAL cells were plated per well (Nalge Nunc International, Rochester, NY, USA) in 200 μl medium (RMPI 1640 with 1% Penicillin G and 5% human serum). Antigens were added in the following concentrations: Phytohaemagglutinin (PHA; (*Hochgereinigtes Phytohämagglutinin HA16*, Order No. R30852801, Oxoid Deutschland GmbH, Wesel, Germany), 5μg/ml; Lipopolysaccharide (LPS; (Bacterial lipopolysaccharide of Salmonella enterica, serotype friedenau H909, was kindly provided by Prof. Dr. H. Brade, Research Center Borstel, Borstel, Germany) 1μg/ml; ESAT-6 (early secretory antigenic peptide -6 from Oxford Immunotec, Abingdon, UK), 10μg/ml; CFP-10 (culture filtrate protein -10 from Oxford Immunotec, Abingdon, UK), 10μg/ml; PPD (Purified protein derivative of tuberculin, Statens Serum Institute, Copenhagen, Denmark), 10μg/ml. PHA and LPS was included as positive controls. After 18 hours, culture supernatants were harvested and frozen at -80°C until analysis.

If sufficient numbers of BAL cells were available, additional infection experiments with three strains of *M*. *tuberculosis* were conducted. Triplicates of 100,000 BAL cells per well were infected at a multiplicity of infection of 5. The strains used were H37Rv (ATCC reference strain), and two Haarlem strains isolated from tuberculosis outbreak clusters, 7761/01 and 9956/03 (personal communication Stefan Niemann, Research Center Borstel). All strains were cultivated under the same conditions and infections were performed in parallel at the same bacterial load. CFUs for each strain were determined in each infection experiment to check the infection rate. Infected cells were incubated in a 48-well plate in 300 μl medium (RPMI with Penicillin G, Amphotericin B and 10% human serum), for 18–24 hours before supernatants were harvested, filtered-sterilised and frozen at -80°C. For logistical reasons, BAL cell antigen stimulations and mycobacterial infections were done separately in distinct laboratories. Therefore, incubation times and experimental conditions for (un)stimulated and (un)infected samples may not be identical (e.g. longer pre-experimental delay due to S3 safety standards, infection experiments were performed in negative pressure laboratories, incubation time ranges 18–24 hours).

Cytokine concentrations in supernatants were measured on a Luminex platform. For every sample, the following 29 cytokines and chemokines were analysed: Eotaxin, G-CSF, GM-CSF, Interferon-α2 (IFN-α2), IFN-γ, Interleukin-10 (IL 10), IL-12p40, IL-12p70, IL-13, IL-15, IL-17, IL-1Rα, IL-1α, IL-1β, IL-2, IL-4, IL-6, IL-7, IL-8, IP-10, MCP-1, MIP-1α, MIP-1β, Tumour necrosis factor-α (TNF-α), VEGF (Millipore human 29-plex kit; Merck, Darmstadt, Germany).

## Data analysis

For several cytokines and under some experimental conditions, values were below the detection limit of the assay, limiting statistical power in such conditions. Cytokines EGF, IL-3, IL-5 and TNF-β were excluded altogether because of insufficient detectable values. Measurements for all other cytokines were natural log-transformed towards normality prior to analysis. For both blood IGRA and BAL IGRA stratifications, separate statistical linear mixed-effect models of log-transformed values were used to estimate the effect of antigen stimulations and infections versus unstimulated and uninfected controls, respectively.

The linear mixed-effects models were adjusted for plate (experimental batch) as fixed effects, while subject and well were included as random effects to adjust for possible correlations between measurements from the same donor and from the same test position. Linear mixed-effect models were also used to estimate the relationship between cytokine level and the proportion of lymphocytes, granulocytes and macrophages in the BAL cells. All graphs and effect sizes, with 95% confidence intervals, were derived from these models. Model parameters are jointly estimated using restricted maximum likehood (REML). All modelling and graphics were done using functions from base R and R packages *effects*, *lmerTest* and *lme4*, available from www.r-project.org [22–24].

## Results

The complete dataset of all analysed subjects is available in the supplementary material (S1 Data).

### BAL cell composition

Since many cytokines are preferentially produced by distinct cell types, we first analysed the composition of cell types in the BAL fluid from each study participant.

**Fig 1:**
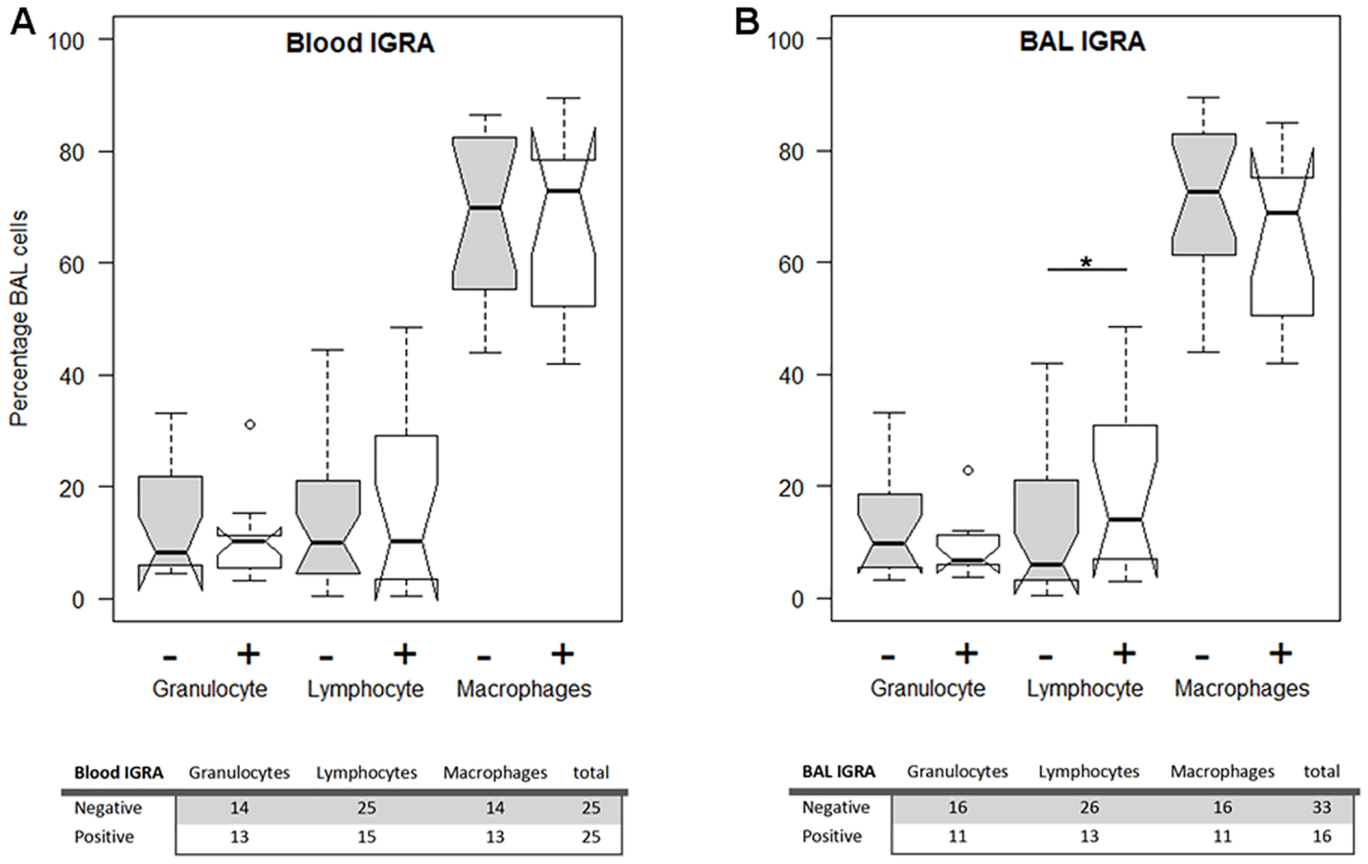
Cellular proportions in the BAL stratified by IGRA. Proportion of the three major cell types in the BAL fluid from study participants, stratified by A) blood and B) BAL IGRA status. Notched boxplots showing five number summaries (minimum, first quartile, median, third quartile, maximum) with outliers. The lower tables indicate the number of donors in each group for which cell counts for the respective cell type were available.

There were no major differences in the proportions of the three most frequent cell types (granulocytes, lymphocytes and macrophages), regardless whether the groups were stratified according to blood IGRA or BAL IGRA results (Fig 1). The only exception was an estimated 8.91 (95%: CI:0.61 to-17.21; p-value = 0.0356) higher percentage of lymphocytes in BAL IGRA-positive compared to BAL IGRA-negative donors.

None of the measured BAL lymphocyte subsets differed significantly when comparing cells from blood IGRA-positive versus -negative individuals except for the tiny subset of regulatory T-lymphocytes which was lower in blood IGRA-negative individuals (0.675 ± 0.385% versus 2.448 ± 1.36%, p<0.0005) which is in accordance to our recent findings [21]. When comparing BAL lymphocyte subsets from BAL IGRA-positive and–negative individuals significantly more CD4^+^CD3^+^ T-lymphocytes and CD4+CD3^+^CD45R0^+^ memory-T-helper lymphocytes were detected in BAL lymphocytes from BAL IGRA-positive individuals (49.57±15.70% versus 37.31±15.56% for CD4^+^ T-lymphocytes, p<0.03; 47.22±16.30% versus 35.11±14.80% for memory T-helper lymphocytes, p<0.02). Neither BAL CD8+ T-lymphocytes nor NK-cells nor naïve lymphocytes differed significantly in BAL IGRA-positive versus BAL IGRA-negative individuals.

### Cytokine induction

**Fig 2:**
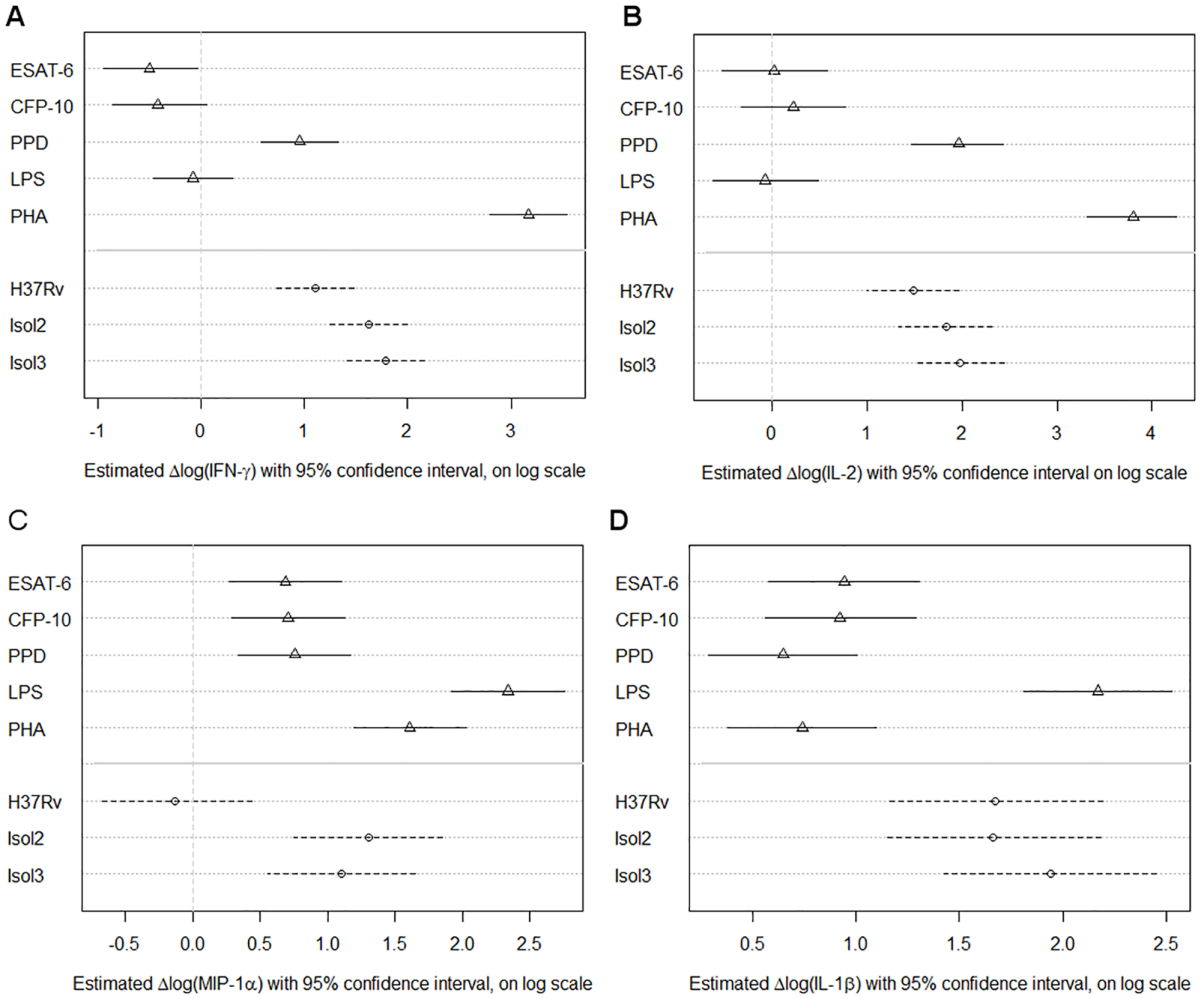
Cytokine induction by antigen stimulation and infection. Induction of cytokines upon activation of lymphocytes and macrophages by antigen stimulation (triangles) and infection (circles). Indicated are natural log-transformed differences between stimulated and unstimulated or infected versus uninfected controls. Symbols show the effect estimates with lines indicating 95% confidence intervals (solid line:antigen stimulation assays; broken line, infection assays). ESAT-6, 6kD Early Secretory Antigenic Target; CFP-10, 10kD Culture Filtrate Protein; PPD, Purified Protein Derivative; LPS, Lipopolysaccharide; PHA, Phytohaemagglutinin; H37Rv, *M*. *tuberculosis* H37Rv; Isol2, *M*. *tuberculosis* isolate 2; Isol3, *M*. *tuberculosis* isolate 3.

There was a wide inter-individual variation of cytokine responses. BAL cells showed clear responses to the positive controls PHA and LPS (Fig 2), indicating viability of the cells. Irrespective of IGRA status, activation of BAL cells upon antigen stimulation and infection was also detected by induction of multiple cytokines. Stimulation with the mycobacterial antigen mixture PPD induced the increased secretion of IFN-γ and IL-2, indicative of lymphocyte activation. Infection of the BAL cells with different *M*. *tuberculosis* strains resulted in a clear induction of mainly macrophage associated cytokines such as MIP-1α and IL-1β (Fig 2 and S1 Table). Remarkably, other chemokines like IL-6, IP-10, MIP-1α and TNF-α, which are also expressed by activated macrophages, were only significantly induced by the two clinical mycobacterial isolates (Isol2 and Isol3), but not by the lab strain H37Rv (S1 Table). Results for all cytokines are summarized in S1 Table listing the p-values for fold changes by each stimulation and infection assay.

### Blood IGRA

The number of donors with a positive or negative blood or BAL IGRA result are summarized in Table 1.

**Table 1:** BAL fluid donors and their IGRA status in either peripheral blood or in BAL cells.

BAL IGRA	Blood IGRA		Total
	Negative	Positive	
Negative	18	14	32
Positive	6	10	16
Unknown	1	1	2
Total	25	25	50

**Fig 3:**
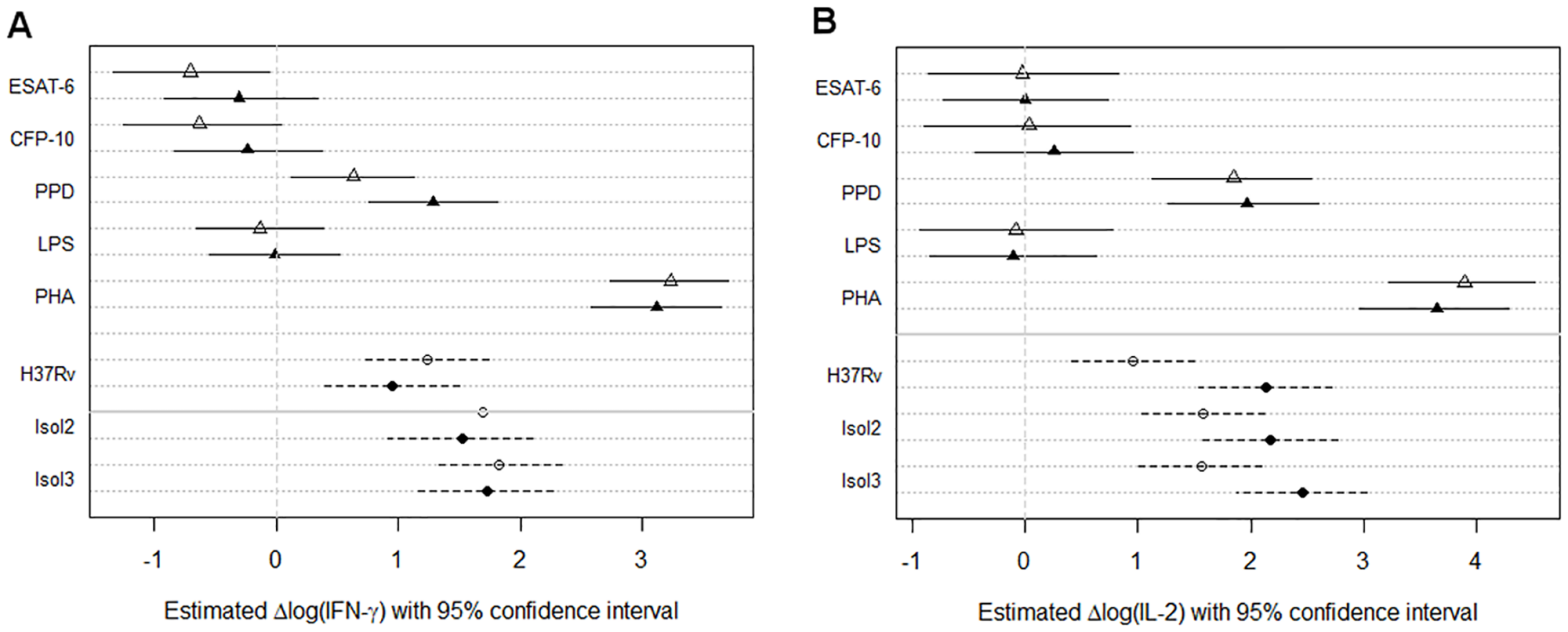
Proinflammatory cytokine induction in blood IGRA negative vs. positive subjects. Induction of IFN-γ and IL-2 by mycobacterial antigens (triangles) and mycobacterial infection (circles), stratified by blood IGRA status (open symbols, IGRA-negative donors; closed symbols, IGRA-positive donors). Plots show the effect estimates with lines (solid line, antigen stimulations; broken line, *M*. *tuberculosis* infection assays) representing 95% confidence intervals. ESAT-6, 6kD Early Secretory Antigenic Target; CFP-10, 10kD Culture Filtrate Protein; PPD, Purified Protein Derivative; LPS, Lipopolysaccharide; PHA, Phytohaemagglutinin; H37Rv, *M*. *tuberculosis* H37Rv; Isol2, *M*. *tuberculosis* isolate 2; Isol3, *M*. *tuberculosis* isolate 3.

There were few correlations of blood IGRA status with cytokine patterns in BAL cell culture supernatants (S1 Fig and S2 Table). All infections triggered an IFN-γ and IL-2 response, whereby cells from blood IGRA-positive donors infected with both H37Rv and *M*. *tuberculosis* Isolate 3 showed a significantly stronger IL-2 response than cells from blood IGRA-negative subjects (Fig 3 and S2 Table). The estimated positive minus negative IGRA effect of infections with *M*. *tuberculosis* H37Rv was 1.18 (0.49 to 1.91; p-value = 0.0019) with *M*. *tuberculosis* Isolate 2 was 0.60 (-0.08 to 1.36; p-value = 0.1069) and with *M*. *tuberculosis* Isolate 3 was 0.90 (0.24 to 1.62; p-value = 0.0131). The higher levels of Eotaxin and IL-7 in antigen and positive control stimulated cells from IGRA-positive donors (S2 Table) are not likely to be a valid effect of the stimulations but may have been caused by experimental bias (e.g. cytokine concentrations close to the detection threshold of the assay), even though most possible confounding experimental factors were adjusted for in the models.

### BAL IGRA

**Fig 4:**
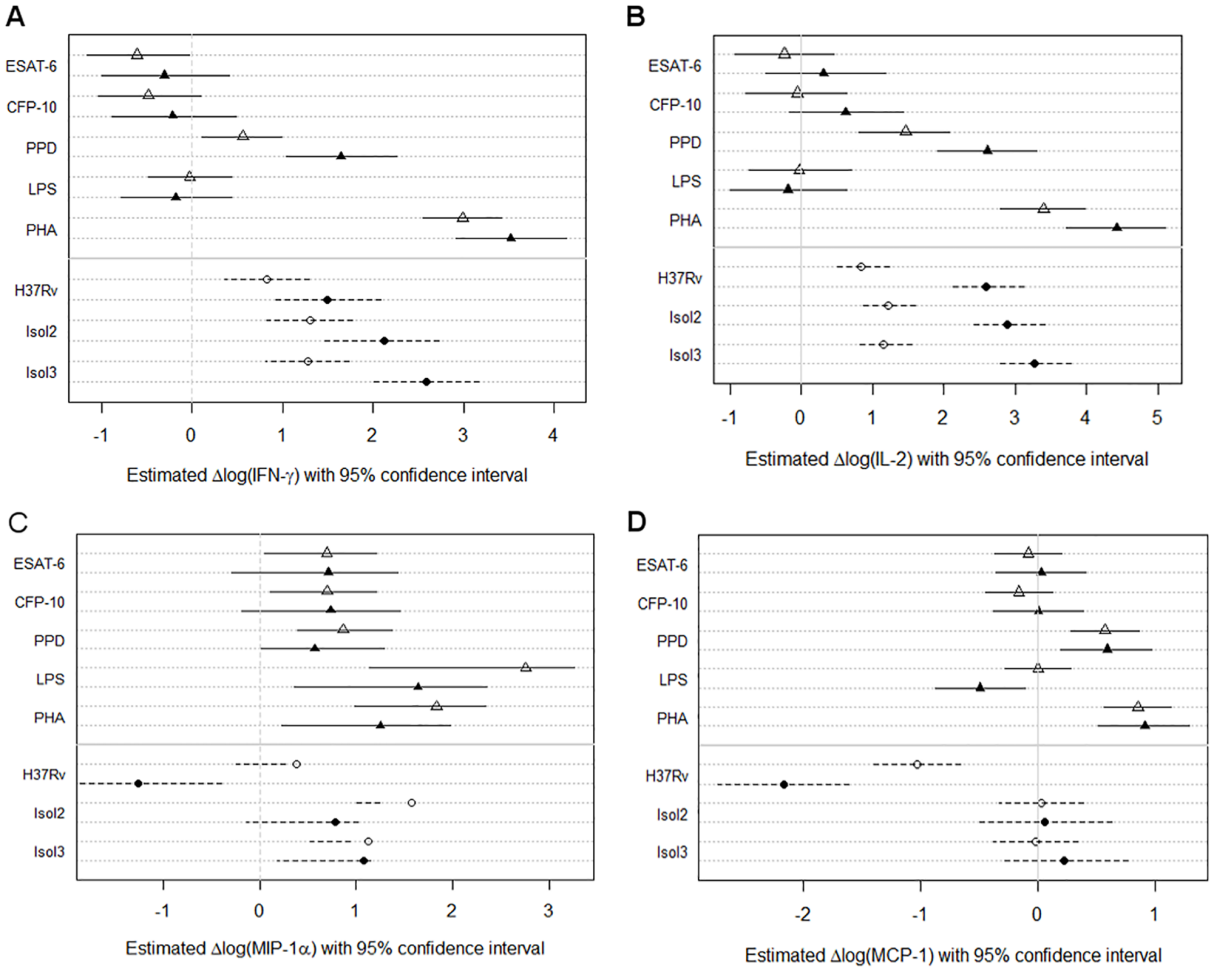
Cytokine induction in BAL cell IGRA negative and positive subjects. Induction of cytokines by activated lymphocytes and macrophages upon antigen stimulation (triangles) or infection (circles), stratified by BAL IGRA status (open symbols, BAL IGRA-negative donors; closed symbols, BAL IGRA-positive donors). The plots show the effect estimates with lines representing 95% confidence intervals (solid line, antigen stimulations; broken line, *M*. *tuberculosis* infection assays). Related p-values are given in S3 Table. ESAT-6, 6kD Early Secretory Antigenic Target; CFP-10, 10kD Culture Filtrate Protein; PPD, Purified Protein Derivative; LPS, Lipopolysaccharide; PHA, Phytohaemagglutinin; H37Rv, *M*. *tuberculosis* H37Rv; Isol2, *M*. *tuberculosis* isolate 2; Isol3, *M*. *tuberculosis* isolate 3.

When analysing the immune response stratified by BAL IGRA results, we did not observe a significant induction of IFN-γ by ESAT-6 or CFP-10 in the cells from IGRA-positive donors, as would have been expected. In contrast, we found several clear correlations with other stimuli or infections, whereby PPD triggered an enhanced IFN-γ (estimated effect 1.09 (95% CI: 0.34 to 1.85); p-value = 0.0063) and IL-2 (estimated effect (1.15 (95% CI: 0.23 to 2.09); p-value = 0.0217) release in BAL IGRA-positive donors (Fig 4 and S3 Table). This enhanced secretion of IL-2 by cells from BAL IGRA-positive donors was even more pronounced in cells infected with *M*. *tuberculosis* (all p-values < 0.0001; estimated differences H37Rv, 1.76 (95% CI: 1.11 to 2.42); Isolate 2, 1.67 (95% CI: 1.03 to 2.35); Isolate 3, 2.11 (95% CI: 1.47 to 2.77).

Intriguingly, only the two clinical *M*. *tuberculosis* strains triggered an increased induction of IFN-γ in BAL IGRA-positive donors (estimated effects for Isolate 2, 0.81 (95% CI: 0.02 to 1.57; p-value = 0.0446) and for Isolate 3, 1.31 (0.55 to 2.04; p-value = 0.0011). In contrast, *M*. *tuberculosis* strain H37Rv seemed to inhibit the secretion of MIP-1α (estimated effect, -1.64 (95%CI: -2.78 to -0.53); p-value = 0.0057) and MCP1 (estimated effect, -1.14 (95%CI: -1.81 to -0.47); p-value = 0.0014) in cells from BAL IGRA-positive individuals only (Fig 4). The same effect was observed for IFN-α2 in H37Rv infected cells (estimated effect, -0.38 (95%CI: -0.65 to -0.11); p-value = 0.0082) and for IL-6 upon LPS stimulation (estimated effect, -1.14 (95%CI: -1.85 to -0.44); p-value = 0.0020). Details are given in S2 Fig and S3 Table. These latter four cytokines and chemokines are all primarily secreted by macrophages.

### Basal cytokine production

**Fig 5:**
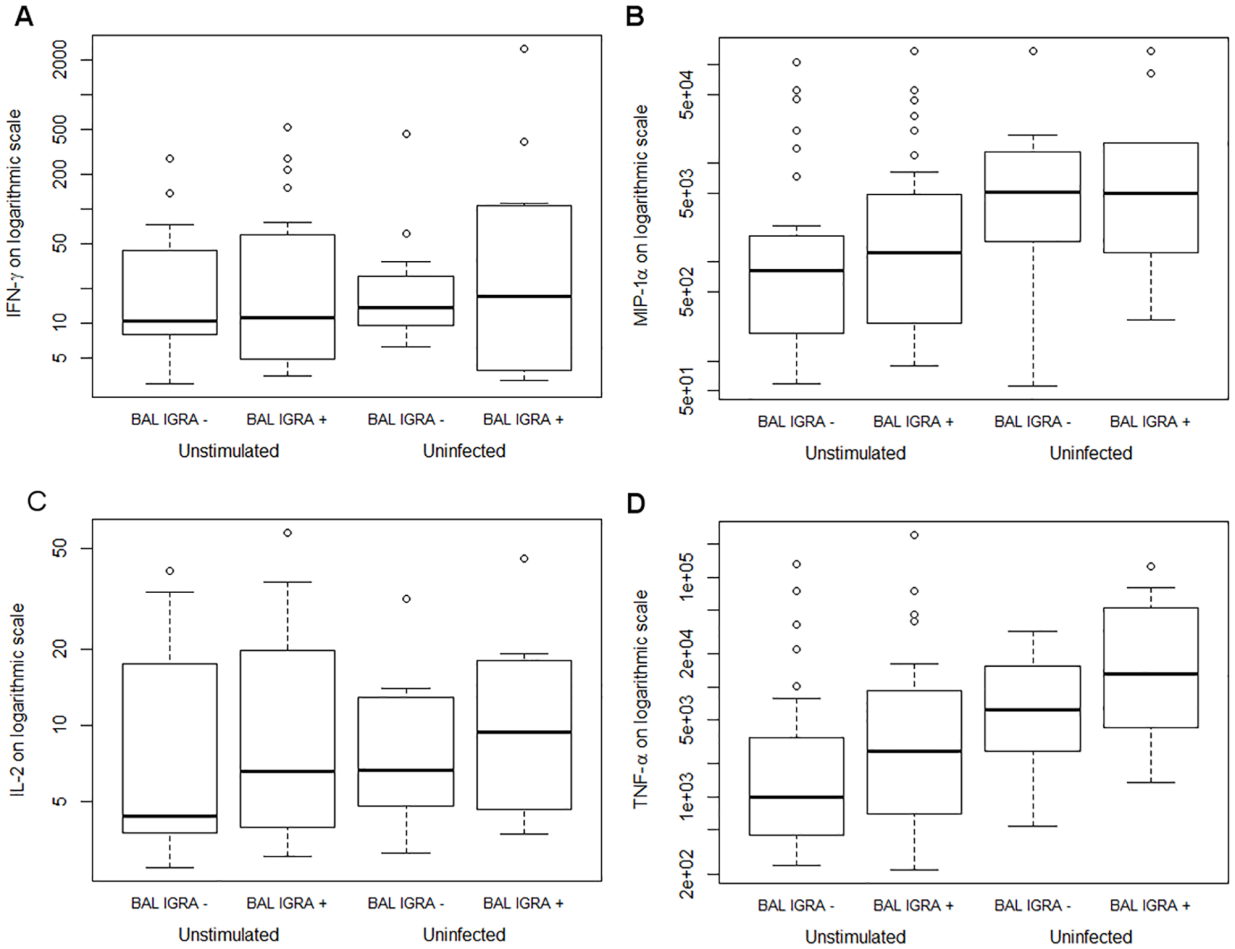
Basal cytokine production in BAL cells. Basal levels of cytokine secretion in unstimulated and uninfected BAL cells stratified by BAL IGRA results. Boxplots showing five-number summaries (minimum, first quartile, median, third quartile, maximum).

Since significant differences between BAL IGRA-positive and negative donors were observed, we determined the basal cytokine levels in the unstimulated and uninfected samples. The stronger IFN-γ and IL-2 secretion in BAL IGRA-positive cells as seen above was not due to higher basal levels in uninfected samples (Fig 5 and S4 Table). Neither was there any difference in MIP-1α levels in uninfected samples. Only for TNF-α consistently different levels were measured in both unstimulated and uninfected control cells from BAL IGRA-negative compared to positive donors (Fig 5).

For a few cytokines, significant differences were observed in BAL IGRA-negative versus positive donors in the unstimulated cells but not in the uninfected cell samples, or vice versa (S4 Table). Note that due to logistical requirements, as mentioned in the materials and methods, the antigen stimulations (including the unstimulated control) and the *M*. *tuberculosis* infections (with their respective uninfected controls) were performed in parallel but in different laboratories. Thus, the results of cytokines with significant differences before infection or stimulation (S4 Table) should be interpreted with caution. We conclude that there were no major differences in the basal cytokine production by BAL cells from BAL IGRA-positive and negative donors.

### Correlations with BAL cell composition

Finally, we analysed basal cytokine levels (i.e. from unstimulated cells) in relation to the proportions of the three main cell types in the BAL, i.e. macrophages, lymphocytes and granulocytes. There were no statistically significant differences between IGRA-positive and IGRA-negative healthy donors in respect to the correlations between proportion of a given cell type and each cytokine (S3 and S4 Figs, stratified by blood IGRA and BAL IGRA, resp.), which is not surprising considering the large variation in BAL cell composition and cytokine responses in relatively small group sizes. However, some trends suggesting cellular pre-stimulation could be observed.

**Fig 6:**
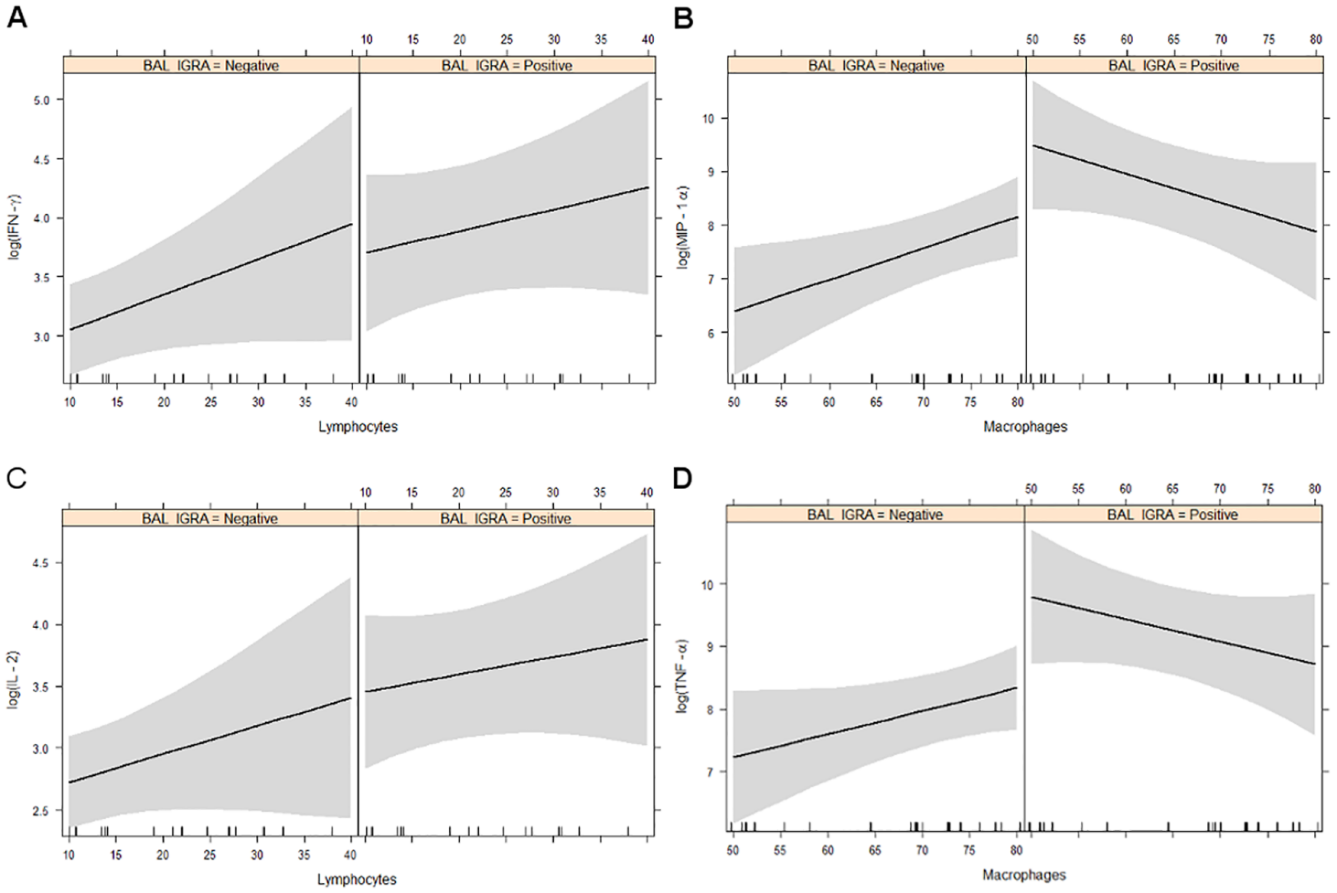
Lymphocyte and macrophage cytokine production in BAL cell IGRA negative and positive subjects. Correlations of cytokine levels with the proportion of lymphocytes and macrophages in the BAL stratified by BAL IGRA result. Left: Estimated linear association between the proportion of lymphocytes and basal IFN-γ and IL-2 levels, with 95% confidence bands, in BAL IGRA-negative and BAL IGRA-positive healthy donors. Right: Correlations between proportion of macrophages and MIP-1α and TNF-α levels.

**Fig 7:**
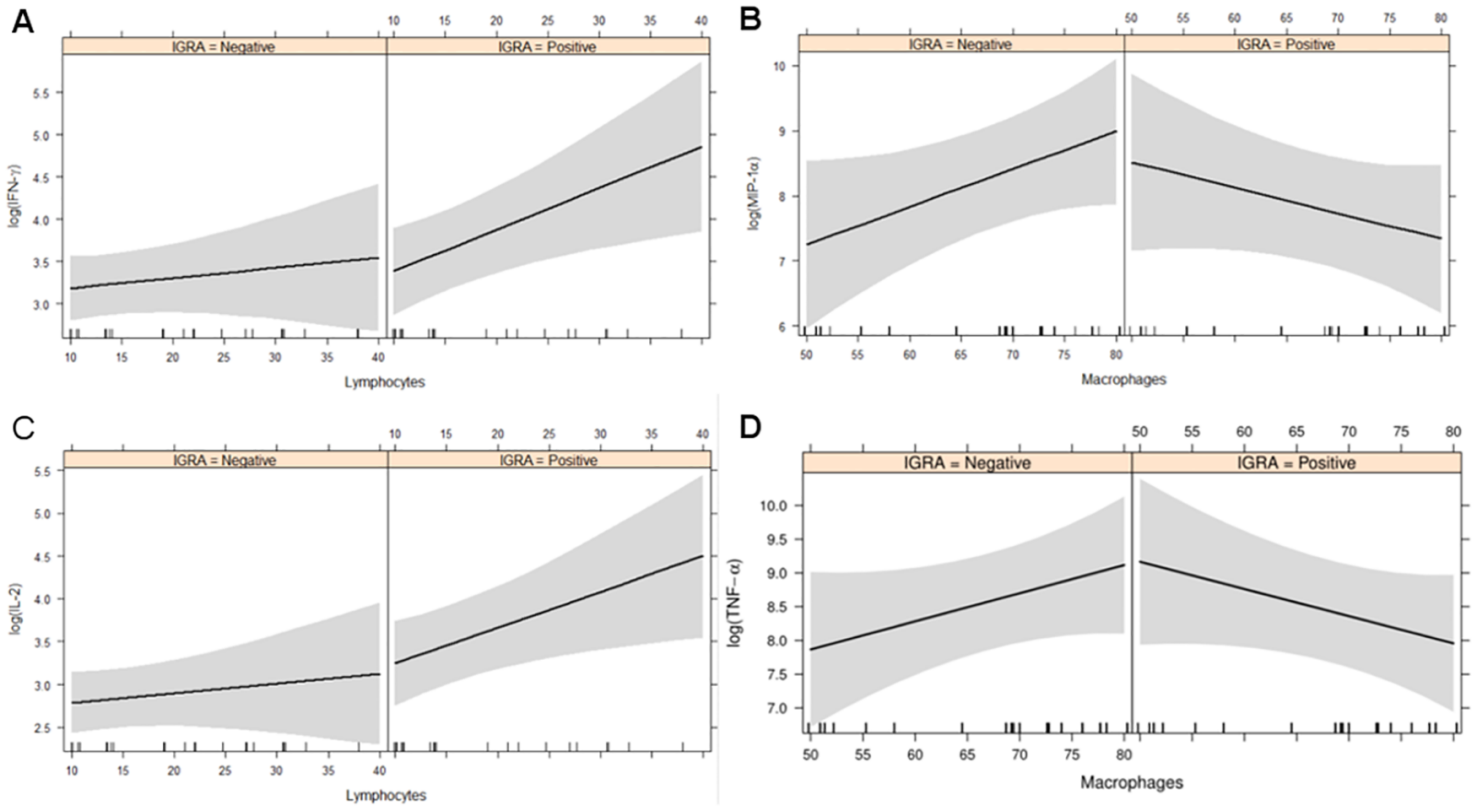
Lymphocyte and macrophage cytokine production in blood IGRA negative and positive subjects. Correlations of cytokine levels with the proportion of lymphocytes and macrophages in the BAL stratified by blood IGRA result. Left: Estimated linear association between the proportion of lymphocytes and basal IFN-γ and IL-2 levels, with 95% confidence bands, in IGRA-negative and IGRA-positive healthy donors. Right: Correlations between proportion of macrophages and MIP-1α and TNF-α levels.

Increasing numbers of lymphocytes in the BAL translated into higher IFN-γ and IL-2 concentrations in both BAL IGRA-positive and BAL IGRA-negative donors (Fig 6a and 6c), pointing to a certain basal secretion of these cytokines in both groups. When stratified by blood IGRA, we observed a steeper increase of these cytokines with increasing lymphocyte proportions (Fig 7a and 7c), which might reflect a higher basal activation state of lymphocytes in the BAL of blood IGRA-positive individuals.

For macrophages, the correlations between cell proportion and cytokine levels revealed unexpected patterns. While concentrations of MIP-1α and TNF-α increased with higher numbers of macrophages in the BAL of blood and BAL IGRA-negative individuals, the opposite was seen in blood and BAL IGRA-positive donors (Figs 6b, 6d, 7b and 7d). These macrophages expressed cytokine patterns reminiscent of cells with immune suppressive phenotype, with higher proportions of macrophages expressing a strong auto-inhibitory effect on cytokine secretion. Alternatively, in BAL samples with low proportions of macrophages, there is a relatively large number of lymphocytes. Pre-activated lymphocytes in the BAL of IGRA-positive individuals might exhibit a stronger stimulation/activation of macrophages at high lymphocyte-to-macrophage ratios. In samples with low lymphocte-to-macrophage ratios stimulatory effects of few lymphocytes were weaker. If this was the case, lymphocytes in the BAL of IGRA-negative donors did not display such a pre-activation state (Figs 6 and 7).

Blood IGRA-positive healthy donors showed a steeper increase in the levels of IL-2 and IFN-γ with increasing proportions of lymphocytes in the BAL, suggesting a higher basal release of these cytokines from BAL cells of blood IGRA-positive individuals (Fig 7).

## Discussion

This study aimed to describe pulmonary cytokine profiles that are associated with the systemic immune responses during LTBI. Our results suggest that individuals with positive IGRA responses in the peripheral blood have pre-activated bronchoalveolar lymphocytes showing an increased response to *M*. *tuberculosis* infection. Furthermore, we detected cytokine patterns indicating enhanced lymphocyte activation but impaired macrophage activation in BAL IGRA-positive individuals. Given the large variations in cytokine secretion and BAL cell composition, a robust pulmonary pattern that could serve as a rationale explaining the development of a systemic immune response could not be defined.

Interleukin-2 is known to play a role in the human immune response to *M*. *tuberculosis* [25]. Few groups have investigated the use of IL-2 levels in BAL samples from patients with tuberculosis and their results do not argue for its use as a biomarker of tuberculosis [26,27]. Our study showed that not only uninfected but *M*. *tuberculosis* infected BAL cells from individuals with LTBI showed a stronger IL-2 response, suggesting general activation of lymphocytes. The most likely, but not yet proven explanation for this observation is a persisting mycobacterial trigger that keeps the human pulmonary immune response in check. In this scenario, increased IL-2 concentrations would enhance lymphocyte proliferation, trigger the secretion of pro-inflammatory molecules, recruit NK cells and indirectly activate macrophages.

However, stratifying our cohort by BAL cell IGRA reactivity allowed a more direct comparison of responses in BAL cells and IGRA reactivity. Individuals with a positive BAL cell IGRA response appear to suffer from macrophage activation. Key cytokines associated with macrophage activation such as MIP-1α or MCP-1 were suppressed upon infection with *M*. *tuberculosis H37Rv* while *M*. *tuberculosis* isolates from clinical outbreaks triggered a stronger response. This might reflect one of many pathomechanisms exploited by *M*. *tuberculosis* during macrophage infection. Mechanisms described include the suppression of PI3Kδ gene expression, modification of miR-26a/KLF4 and CREB-C/EBPβ signalling pathways and others that eventually regulate M1/M2 polarization of macrophages [28,29].

Accordingly, macrophage activation and inflammatory responses are influenced by the mycobacterial strain [30]. Macrophage activation, has been suggested to mirror one aspect of virulence [31]. A recent study on tuberculosis contacts described a T-cell suppressive role of myeloid-derived suppressor cells underlining the importance of the early interplay between innate and adaptive immunity [32]. This can also impact on the cytokine profile following infection of BAL cells with various isolates. Of course, even though infections with the different strains were done in parallel at the same infectious dose, we cannot exclude possible differences in infection efficiency by the different strains.

Alternatively, the differences in cellular activation of BAL cells may be related to the total *M*. *tuberculosis* exposure of the study subjects over time. At least for *M*. *bovis BCG*, it has been shown that macrophage activation—quantified by MIP-1α and MCP-1—depends on the dose of infection [33]. As this cannot be measured in a clinical setting, we standardized the study population by applying a minimal cumulative exposure threshold.

Our findings indicate that probably both, innate responses of the host and *M*. *tuberculosis* virulence factors contribute to divergent activation patterns [34–36]. Importantly, the effects were limited to BAL cell IGRA-positive subjects in our study, suggesting that various features of *M*. *tuberculosis* infection are compartmentalized and thus cannot be observed in the blood.

In general, the experimental setting using *ex-vivo* BAL cells for *in vitro* stimulation is a promising tool that complements our knowledge derived from blood based assays. Cells remained responsive to stimuli, indicating viability. Stimulation of BAL cells with the mycobacterial antigen mixture PPD and infection with different *M*. *tuberculosis* strains induced secretion of multiple pro-inflammatory cytokines and chemokines. Proinflammatory cytokine release following PPD stimulation was in line with pulmonary cellular responses after *in-vivo* and *in-vitro* PPD exposure [13,37,38].

Nevertheless, there were limitations as the BAL cell composition showed large variations among the 50 individuals included. Such variations are common in analyses of BAL fluid [39]. The abundance of several cytokines was low in supernatants and sometimes below the detection threshold. Cytokines in very low concentrations are more prone to false positive signals thus skewing the data. Furthermore, for some BAL IGRA positive individuals, IFN-γ concentrations in culture supernatants from ESAT6 or CFP-10 stimulated cells may remain below the detection threshold even though enough IFN-γ secreting BAL cells could be detected to determine BAL cell IGRA positivity.

Cellular interplay in the BAL compartment is complex and BAL cells from tuberculosis patients display altered expression of genes regulating cytokines and chemokines [40]. In human BALs, numerous studies identified variable patterns of cytokines and chemokines for which an association with pulmonary tuberculosis has been postulated [10,26,41–43]. However, there is scarce information about immune responses of BAL cells to mycobacterial antigens and live *M*. *tuberculosis* infection.

In conclusion, systemic positive IGRA responses did not reflect local immune responses in pulmonary cells obtained by BAL. Divergent cytokine patterns between clinical and laboratory bacterial isolates were observed suggesting an impact of the *M*. *tuberculosis* strain on macrophages activation. This reflects the complex and incompletely understood cellular responses to antigens and mycobacteria. The differing states of activation or suppression of lymphocytes and macrophages in BAL cells from BAL IGRA-positive individuals will need to be confirmed and studied in more detail in further studies. Our findings, thus, add yet another layer of complexity to an already very faceted picture of cellular responses to *M*. *tuberculosis* in the human lung.

## Supporting information

S1 Data Complete dataset of all analysed study subjects. (XLSX)

S1 Fig All cytokines per blood IGRA (negative white symbols, positive black symbols). (DOCX)

S2 Fig All cytokines per BAL IGRA (negative white symbols, positive black symbols). (DOCX)

S3 Fig Associations of BAL cell composition and cytokine concentrations stratified by host blood IGRA. Estimates, with 95% confidence bands, from linear mixed-effects models of association between three cell types (A—granulocytes, B—lympocytes, C—macrophages) and cytokine concentration, stratified by blood IGRA.
(DOCX)

S4 Fig Associations of BAL cell composition and cytokine concentrations stratified by BAL IGRA. Estimates, with 95% confidence bands, from linear mixed-effects models of association between three cell types (A—lympocytes, B—macrophages) and cytokine concentration, stratified by BAL IGRA.
(DOCX)

S1 Table P-values for changes of cytokine concentrations in BAL cell supernatants following antigen stimulation or *in vitro* infection with M. tuberculosis stains. (DOCX)

S2 Table P-values for cytokine concentration differences in BAL cell culture supernatants between blood IGRA-positive and negative subjects. (DOCX)

S3 Table P-values for cytokine concentration differences in BAL cell culture supernatants between BAL IGRA-positive and negative subjects. (DOCX)

S4 Table P-values for cytokine concentration differences in unstimulated versus infected BAL cell culture supernatants at baseline. (DOCX)
