# Supplementary material for: Pulmonary immune responses to Mycobacterium tuberculosis in exposed individuals
Source: PLoS One. 2017 Nov 10;12(11):e0187882. doi: 10.1371/journal.pone.0187882 (PMC5695274; doi:10.1371/journal.pone.0187882)
Supplement: S2 Fig — (DOCX) [file pone.0187882.s003.docx]

**IGRA status and pulmonary immune responses to *Mycobacterium tuberculosis* in exposed individuals**

Christian Herzmann, Martin Ernst, Christoph Lange, Steffen Stenger, Stefan Kaufmann, Norbert Reiling, Tom Schaberg, Lize van der Merwe, Jeroen Maertzdorf for the Tb or not Tb consortium

**Supplementary figure 2**

| **Fig S2.** All cytokines per BAL IGRA (negative white symbols, positive black symbols) | |
| --- | --- |
| 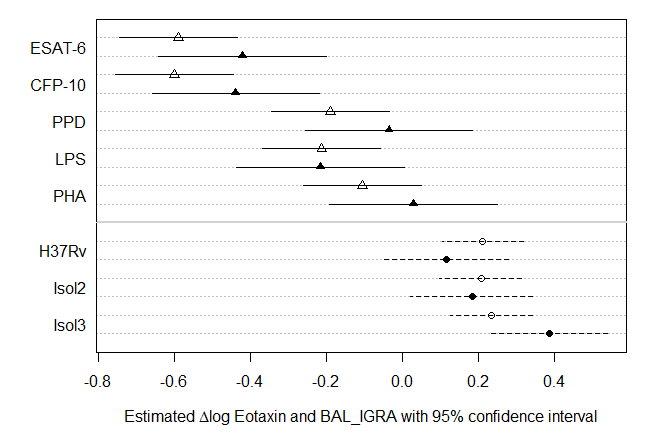 | 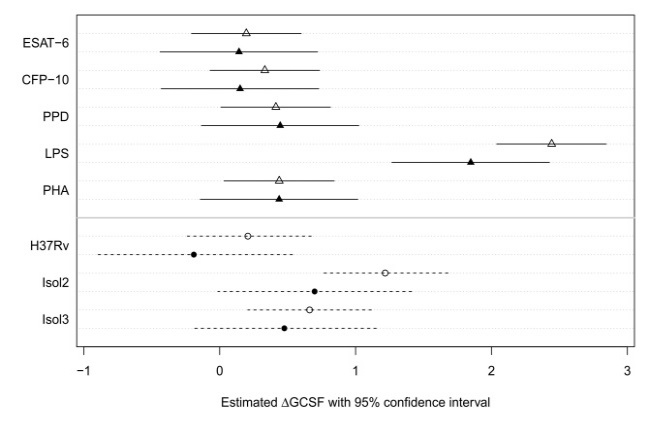 |
| 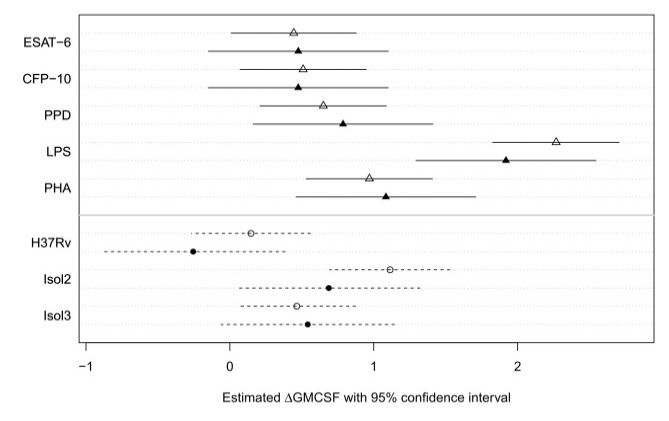 | 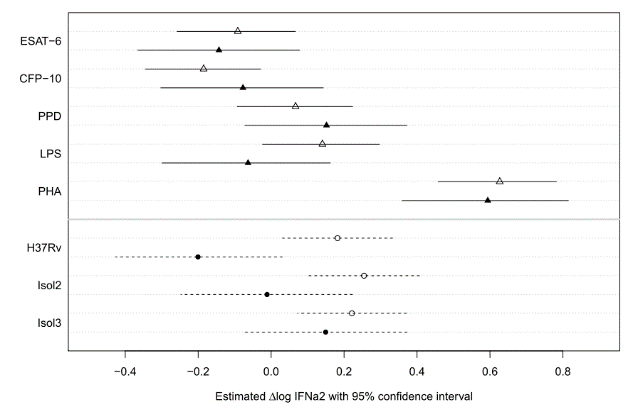 |
| 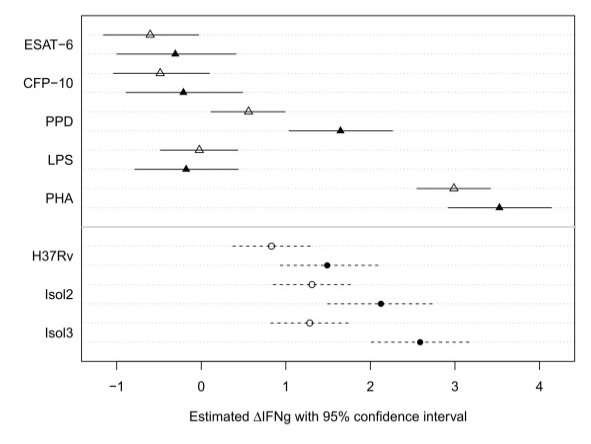 | 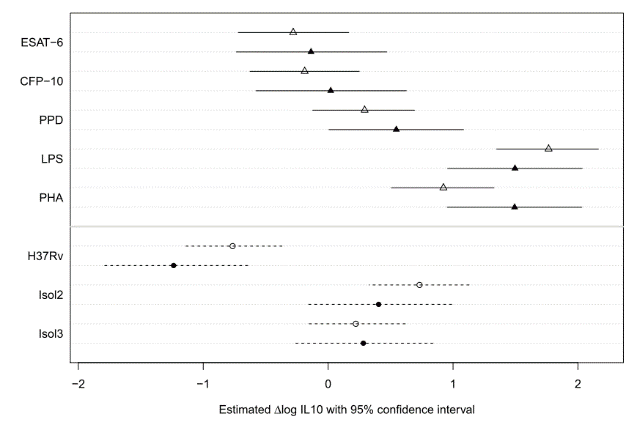 |
| 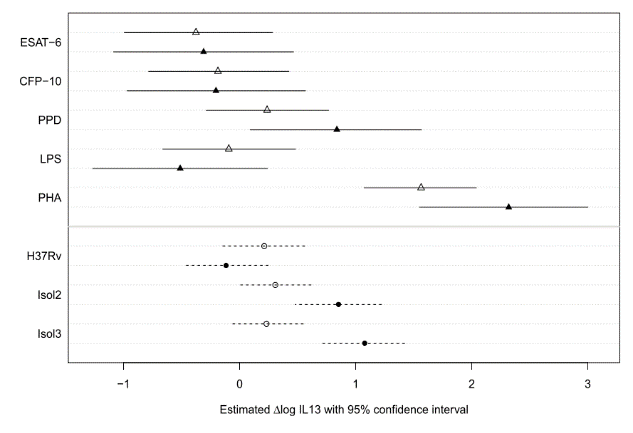 | 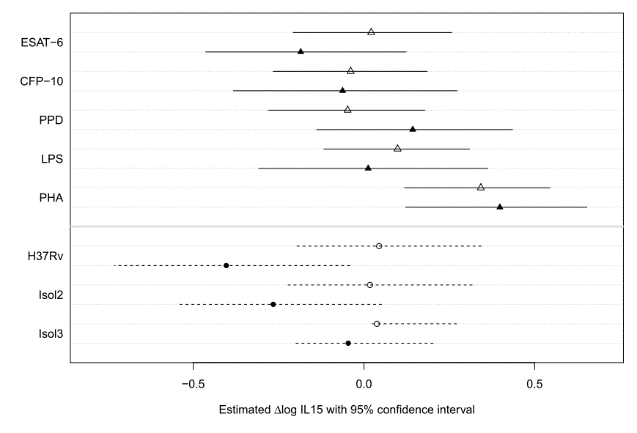 |
| 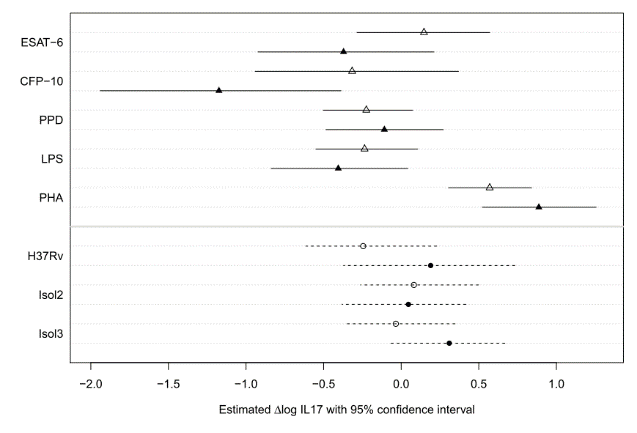 | 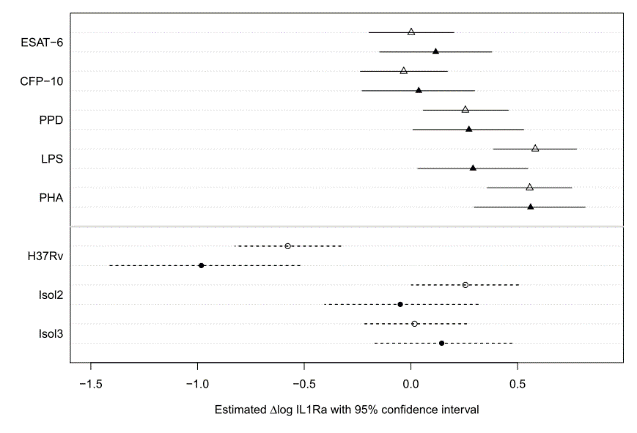 |
| 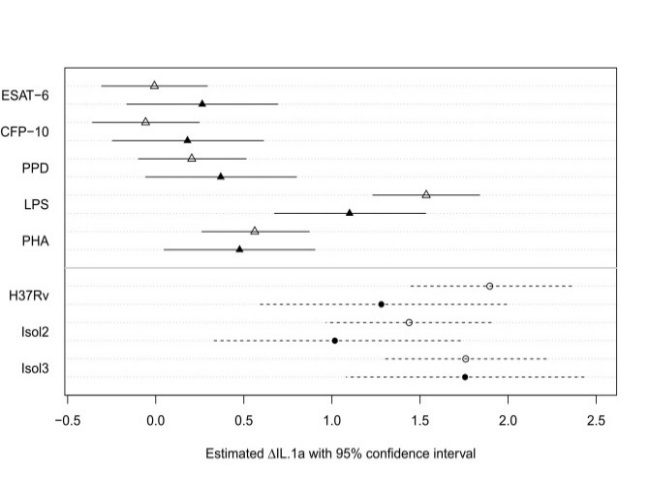 | 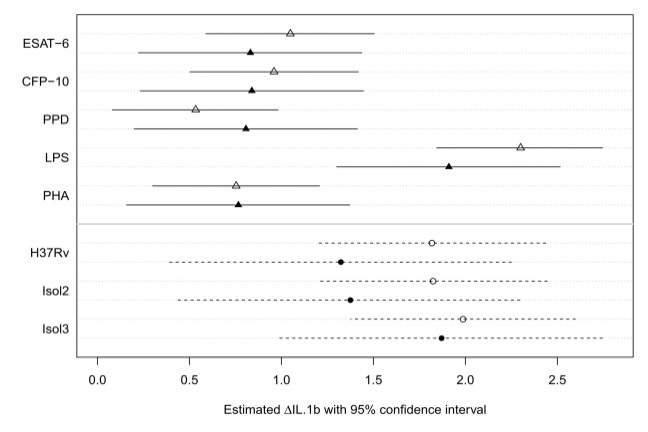 |
| 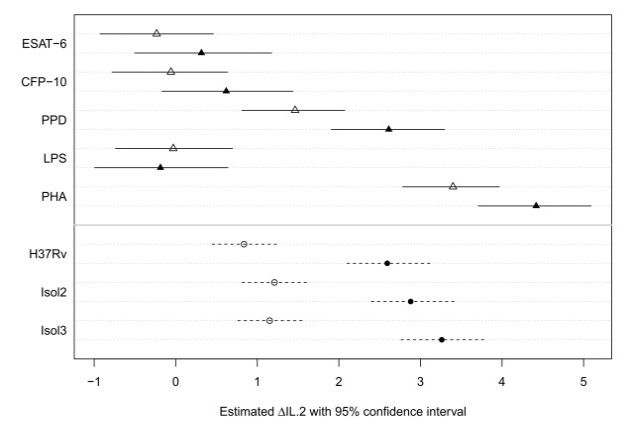 | 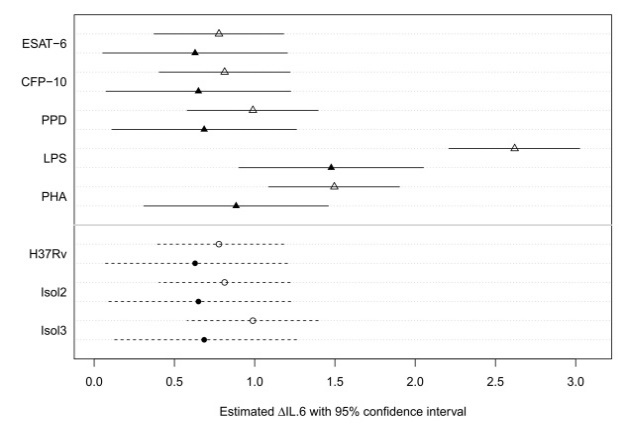 |
| 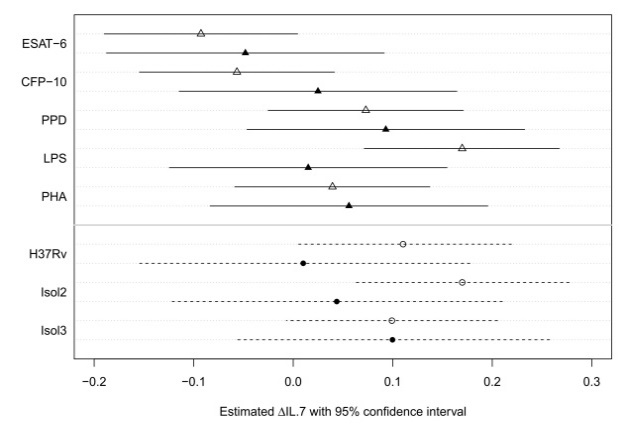 | 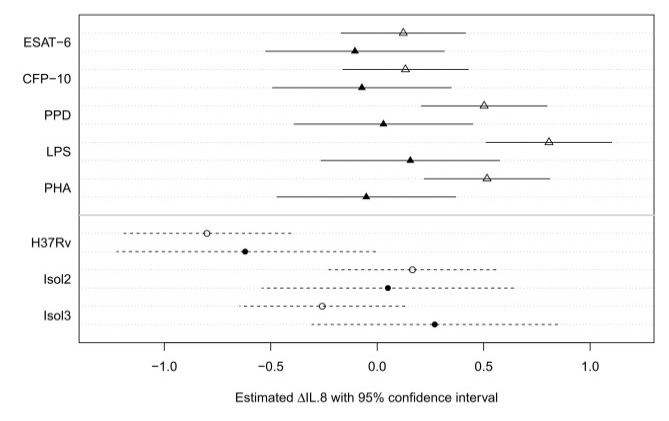 |
| 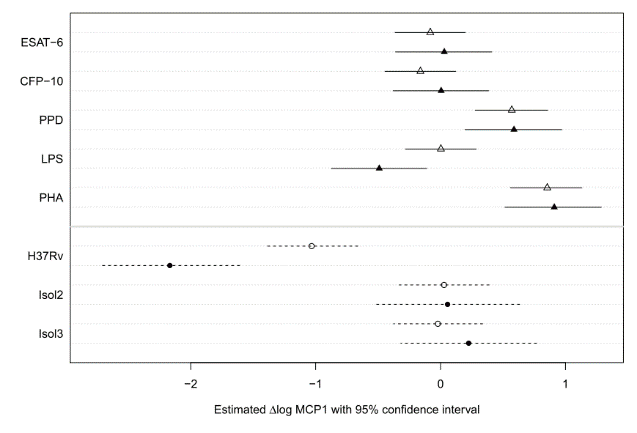 | 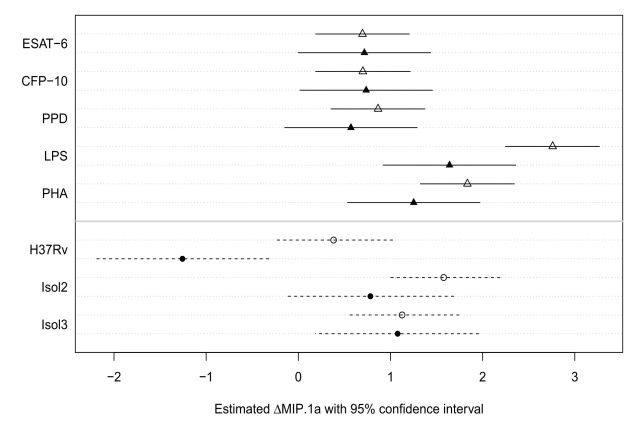 |
| 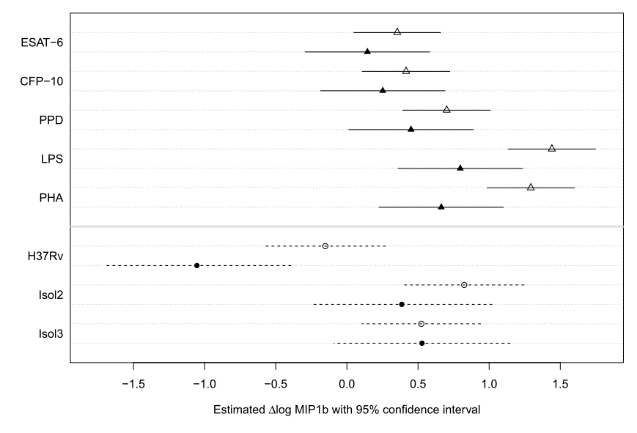 | 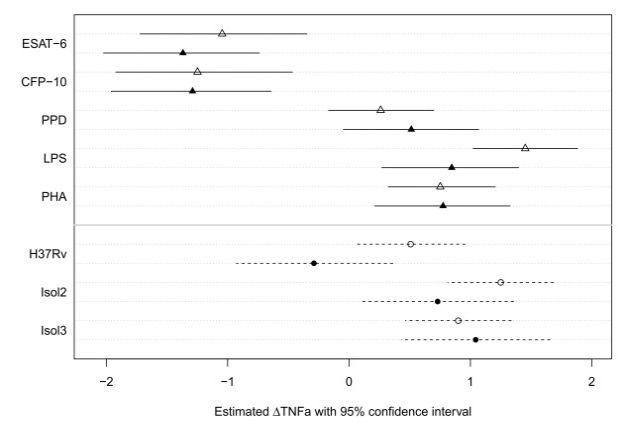 |
| 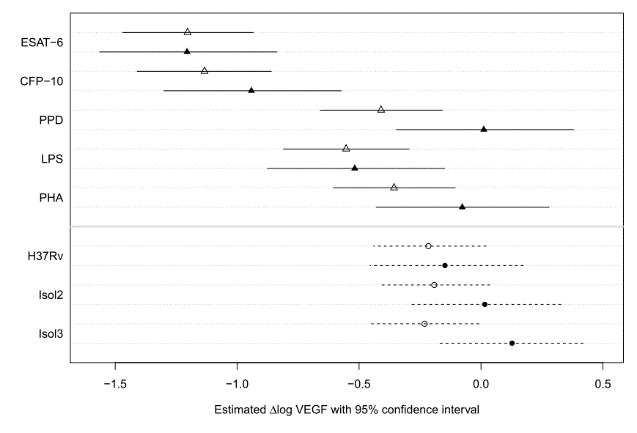 |  |
