# Supplementary material for: Pulmonary immune responses to Mycobacterium tuberculosis in exposed individuals
Source: PLoS One. 2017 Nov 10;12(11):e0187882. doi: 10.1371/journal.pone.0187882 (PMC5695274; doi:10.1371/journal.pone.0187882)
Supplement: S3 Fig — Estimates, with 95% confidence bands, from linear mixed-effects models of association between three cell types (A—granulocytes, B—lympocytes, C—macrophages) and cytokine concentration, stratified by blood IGRA. (DOCX) [file pone.0187882.s004.docx]

**IGRA status and pulmonary immune responses to *Mycobacterium tuberculosis* in exposed individuals**

Christian Herzmann, Martin Ernst, Christoph Lange, Steffen Stenger, Stefan Kaufmann, Norbert Reiling, Tom Schaberg, Lize van der Merwe, Jeroen Maertzdorf for the Tb or not Tb consortium

**Supplementary figure 3**

| **Fig S3A-C.** Estimates, with 95% confidence bands , from linear mixed-effects models of association between three cell types and cytokine concentration, stratified by blood IGRA. | |
| --- | --- |
| **Fig S3A.** Granulocyte associated cytokine concentration stratified by blood IGRA | |
| 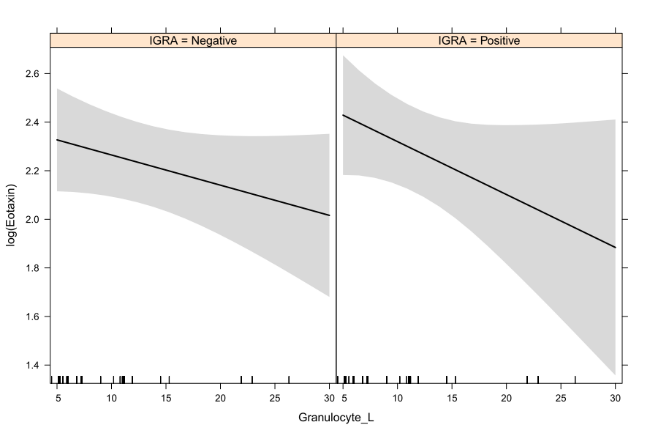 | 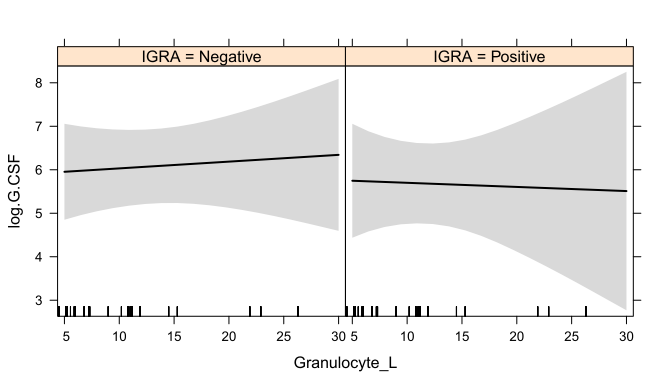 |
| 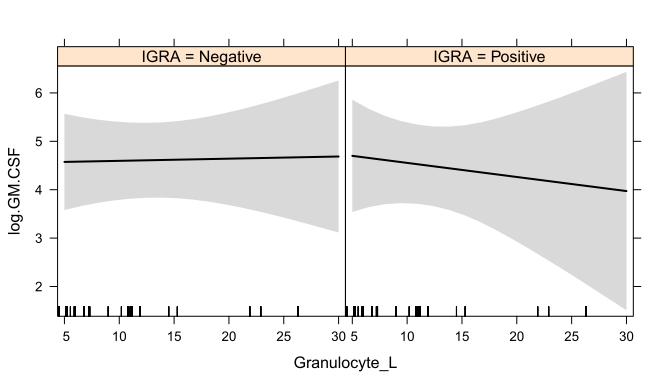 | 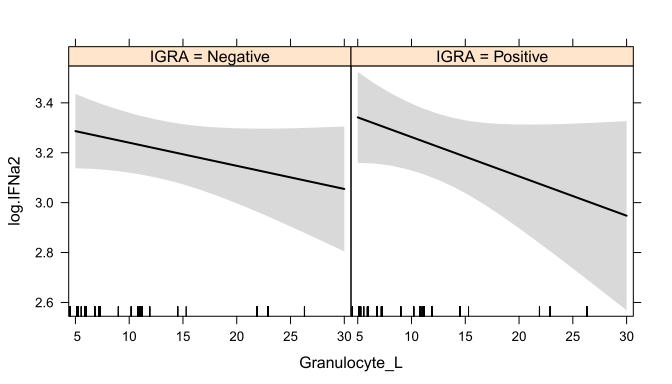 |
| 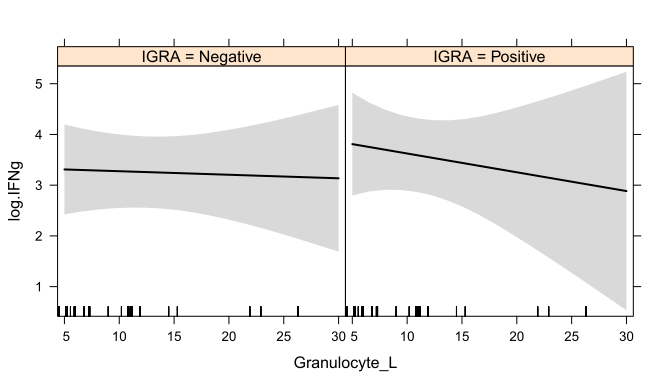 | 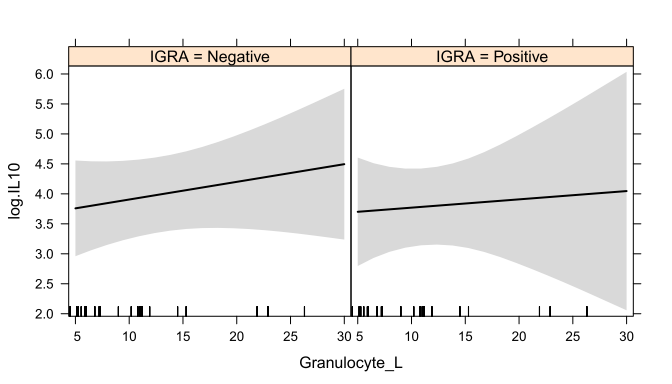 |
| 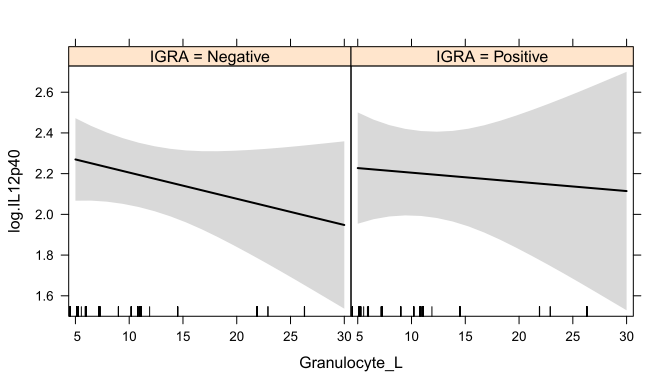 | 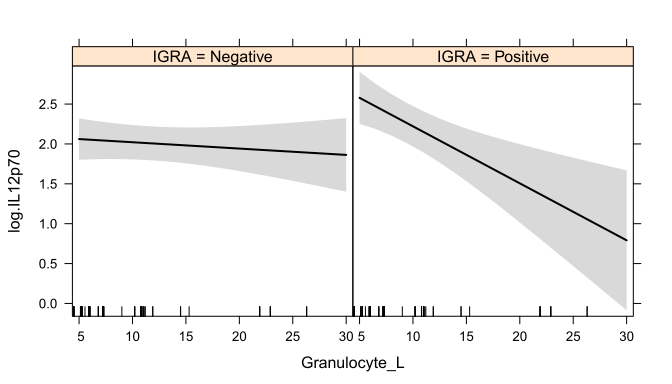 |
| 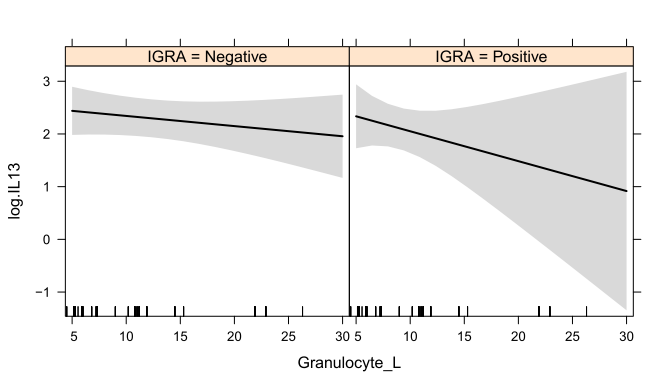 | 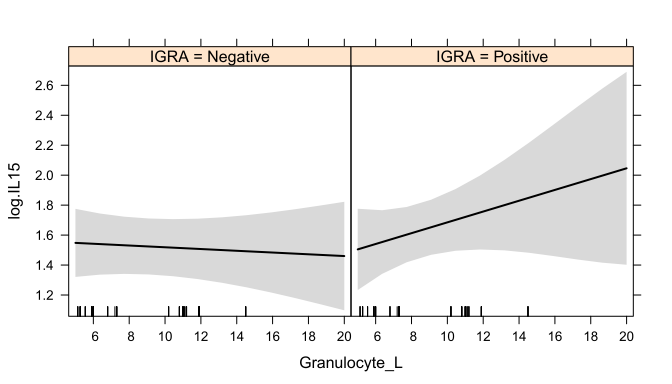 |
| 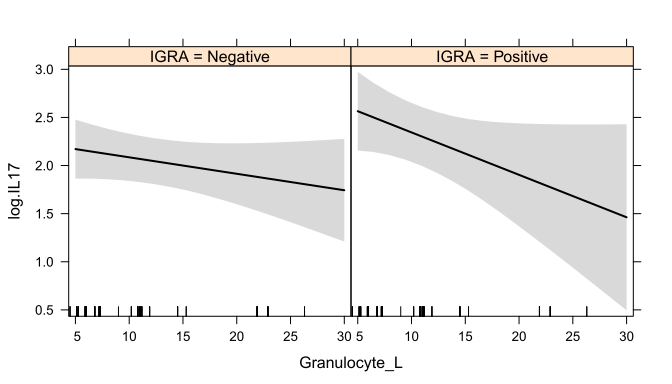 | 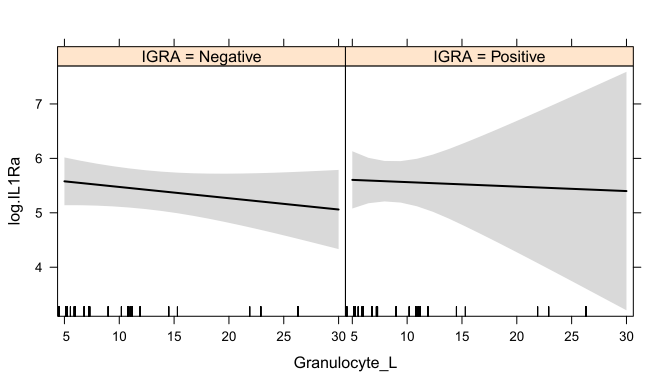 |
| 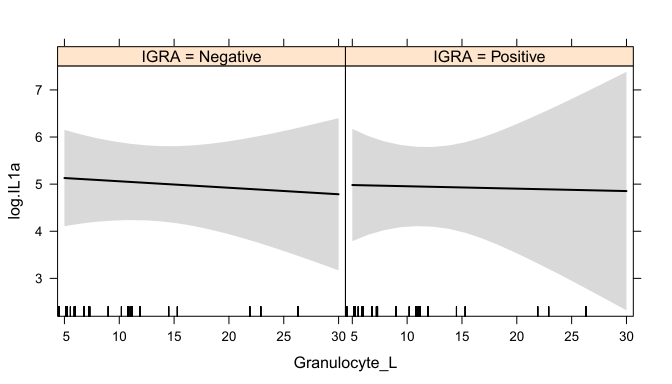 | 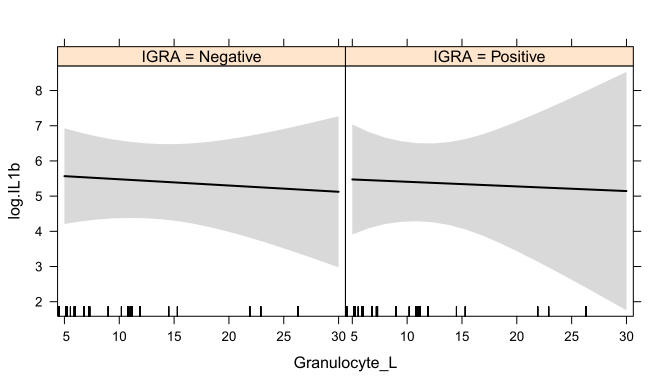 |
| 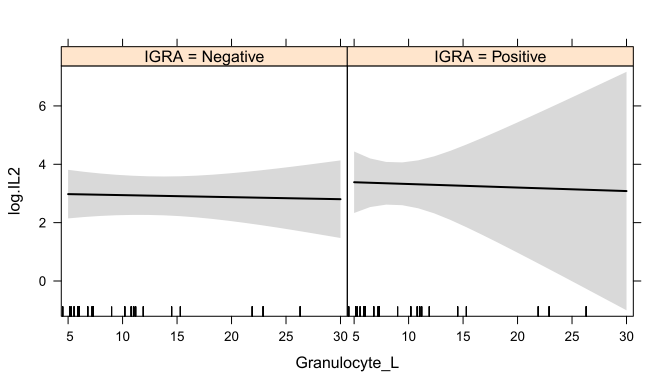 | 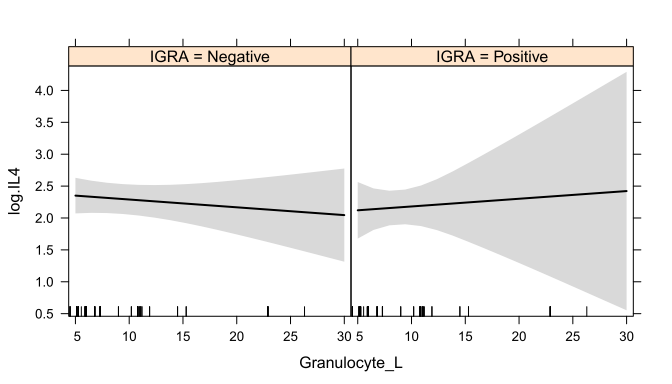 |
| 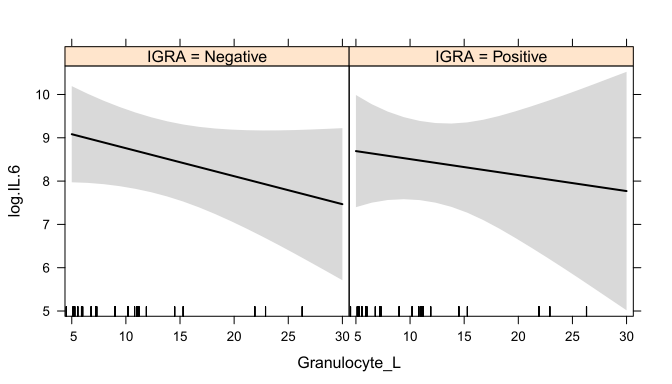 | 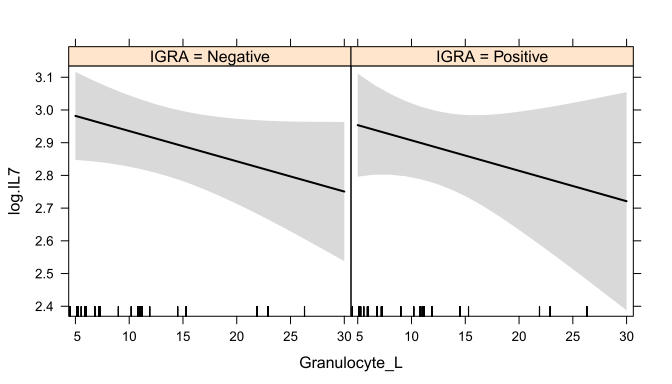 |
| 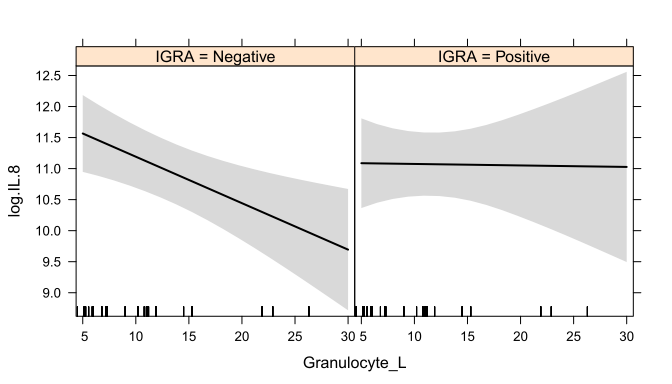 | 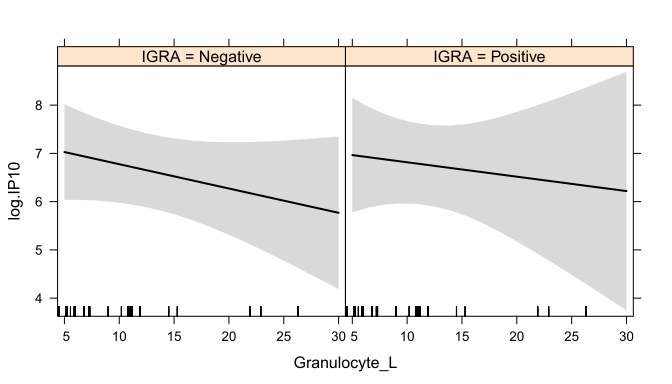 |
| 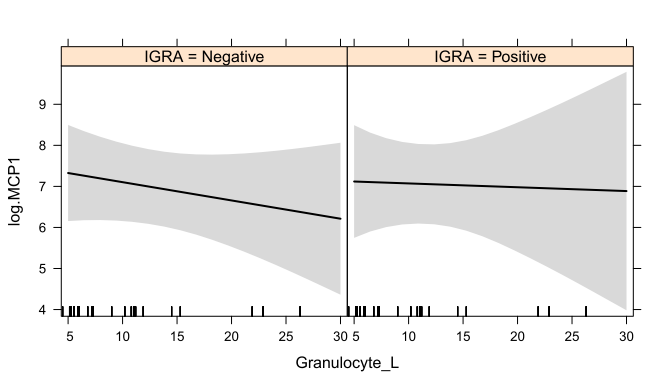 | 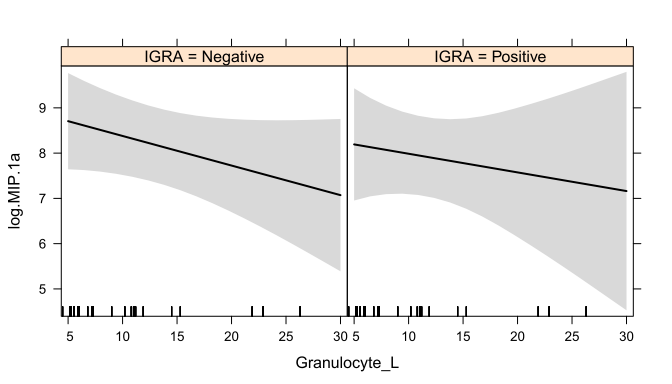 |
| 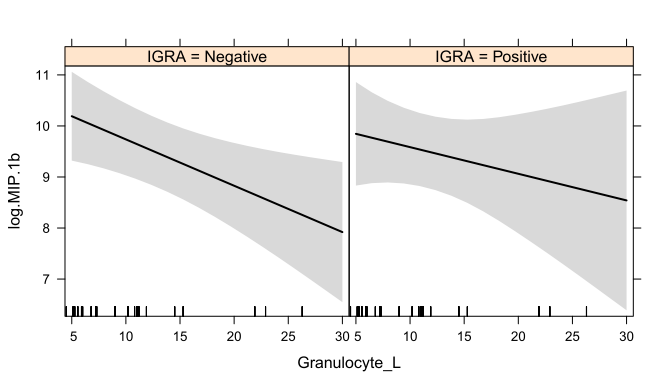 | 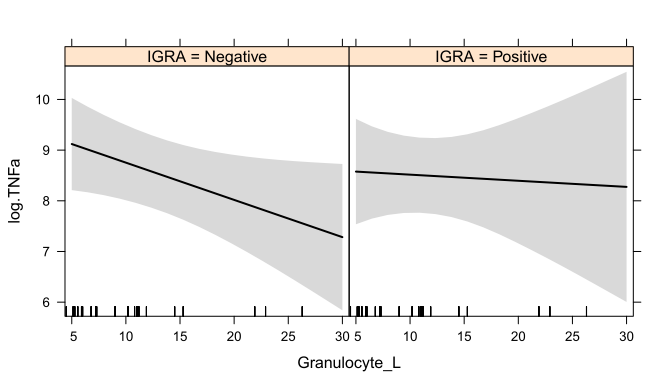 |
| 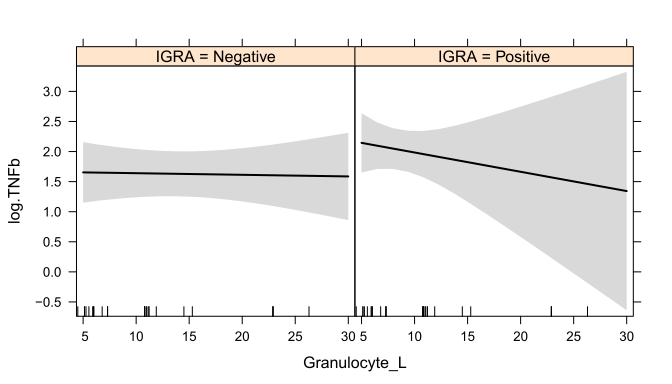 | 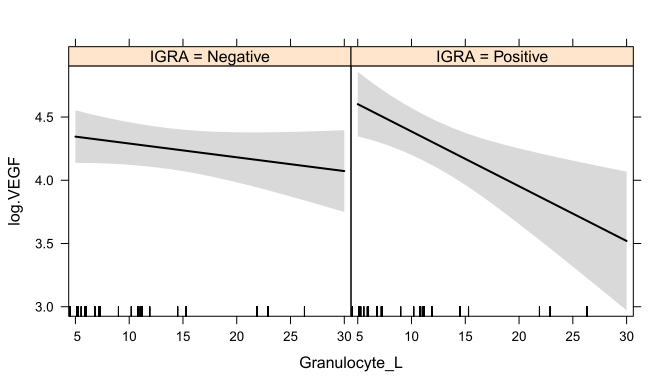 |

| **Fig S3B.** Lymphocyte associated cytokine concentrations stratified by IGRA | |
| --- | --- |
| 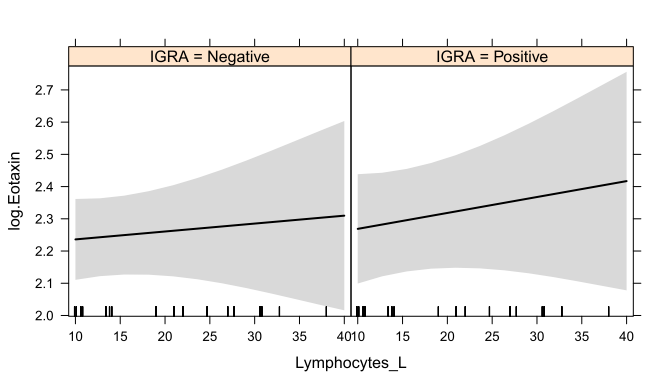 | 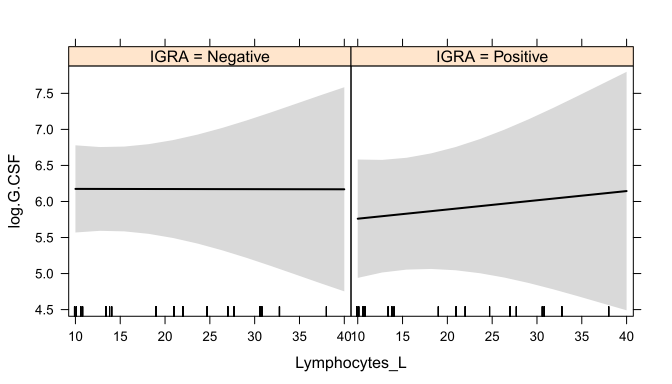 |
| 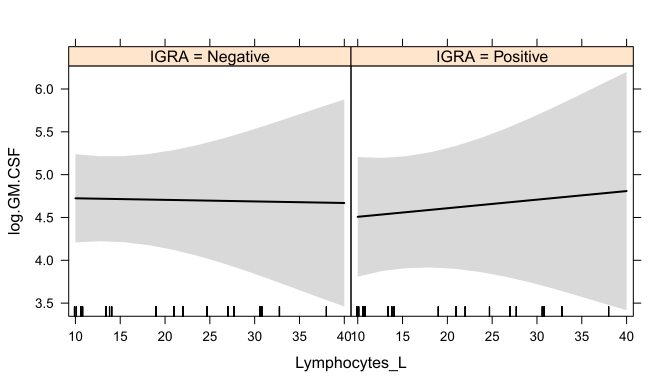 | 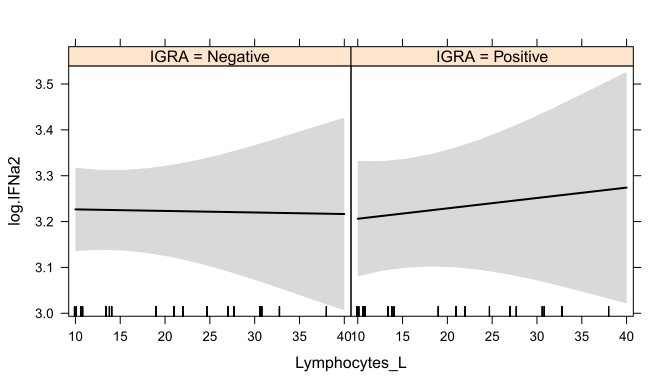 |
| 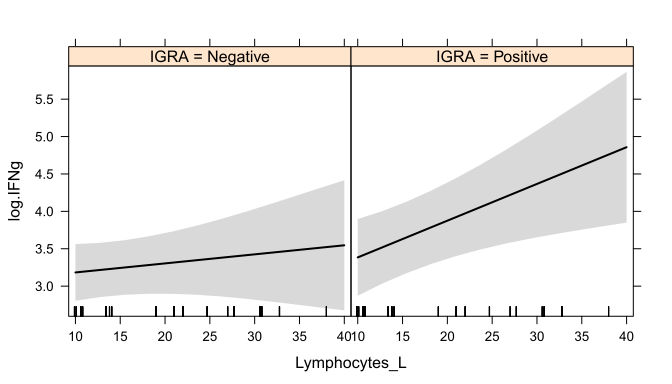 | 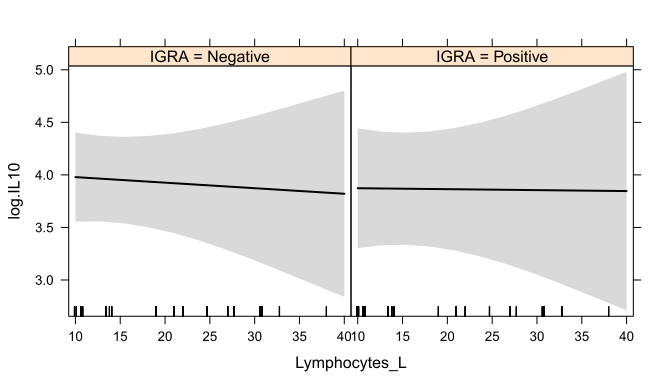 |
| 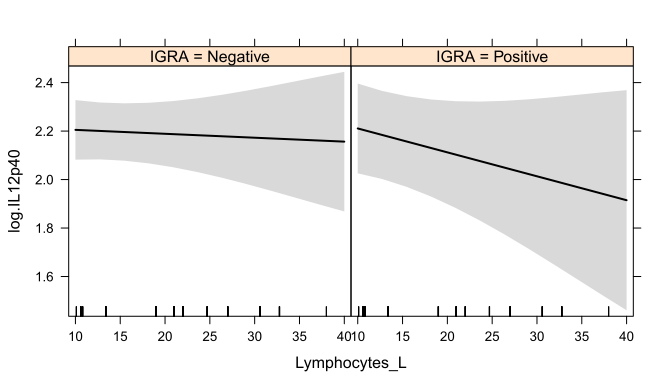 | 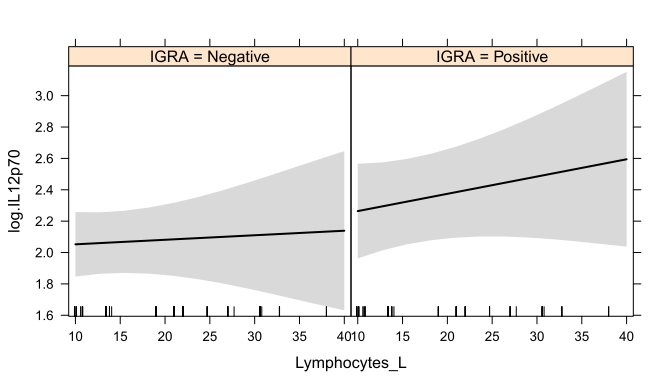 |
| 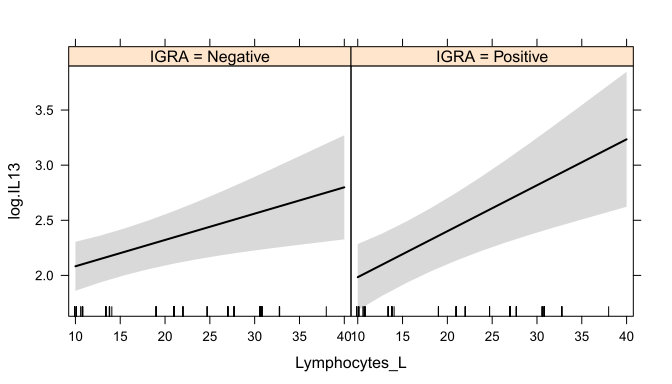 | 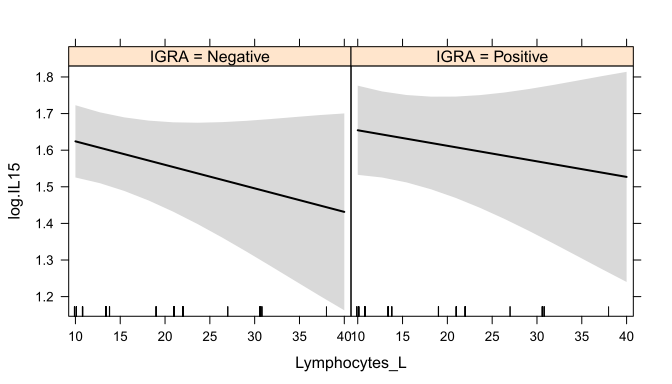 |
| 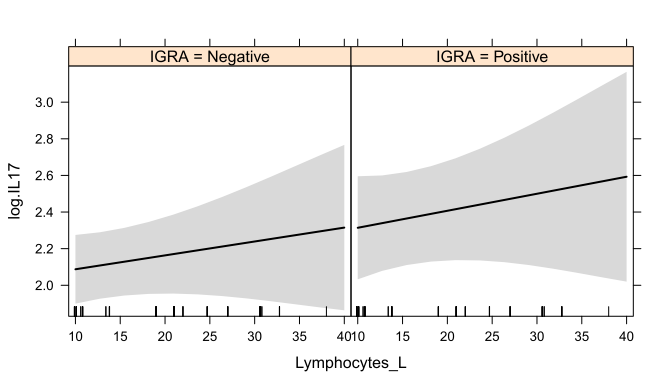 | 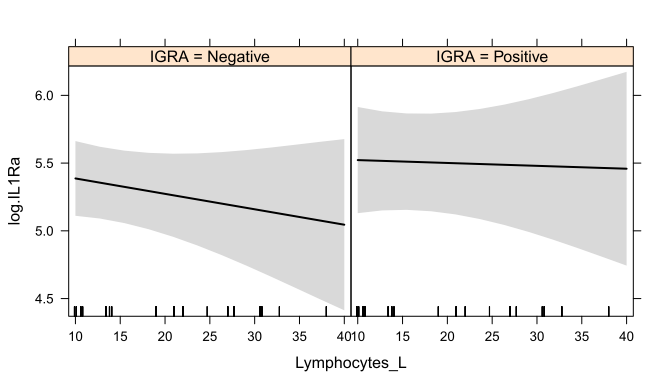 |
| 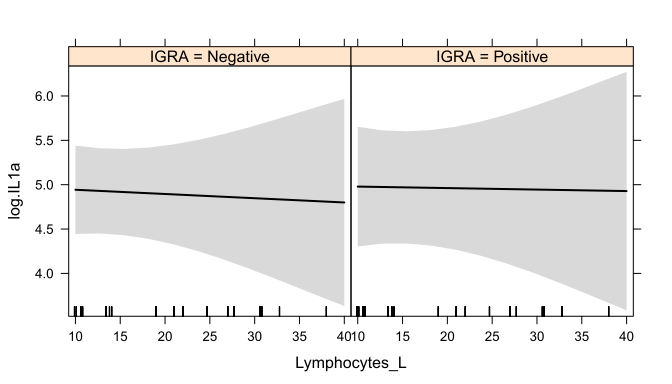 | 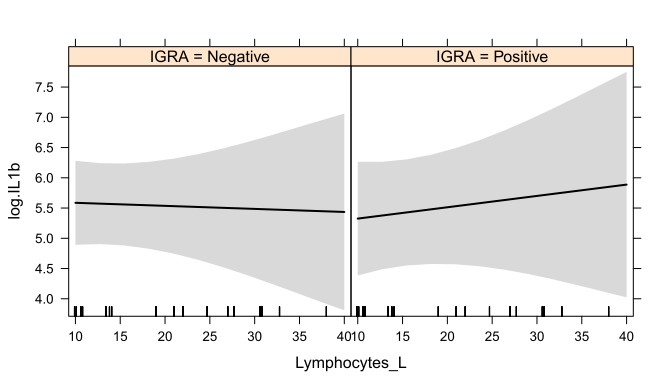 |
| 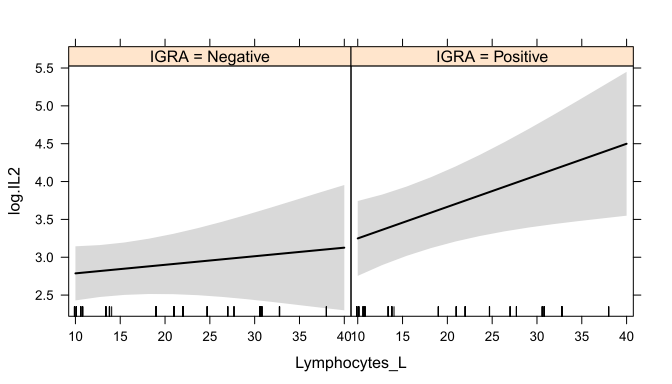 | 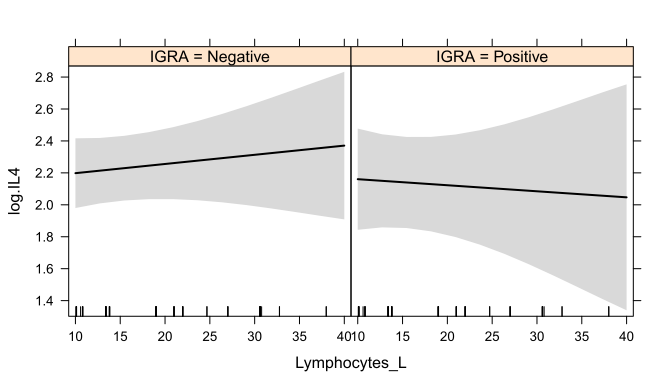 |
| 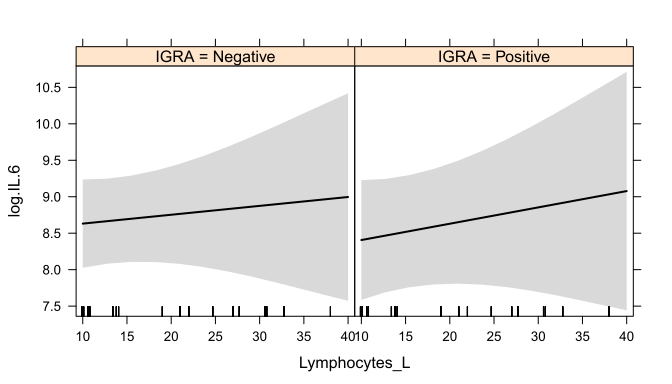 | 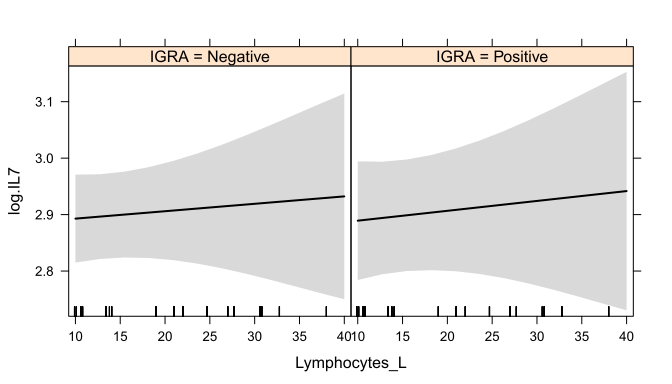 |
| 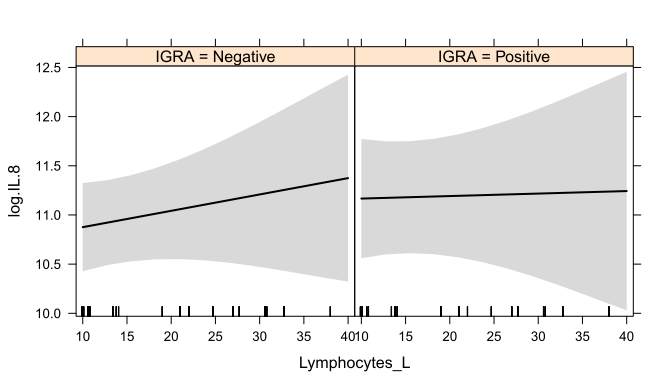 | 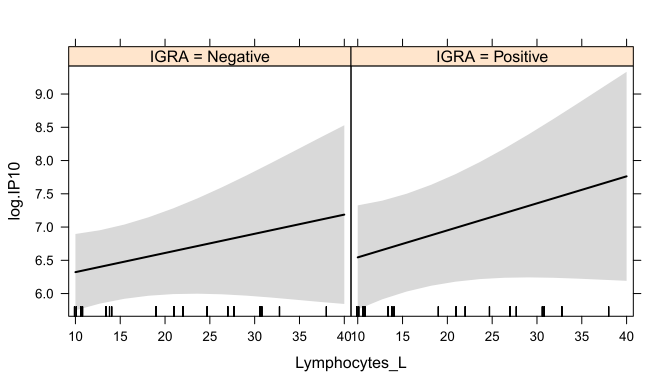 |
| 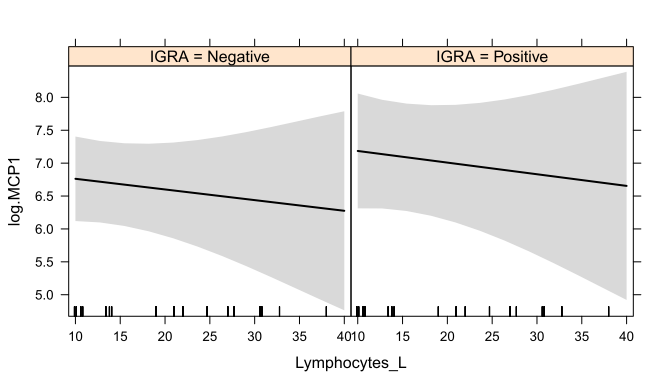 | 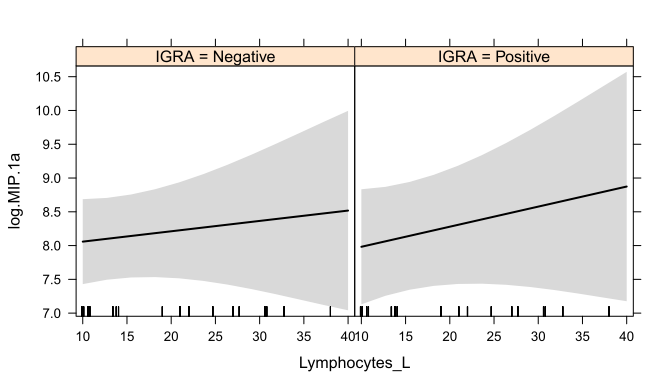 |
| 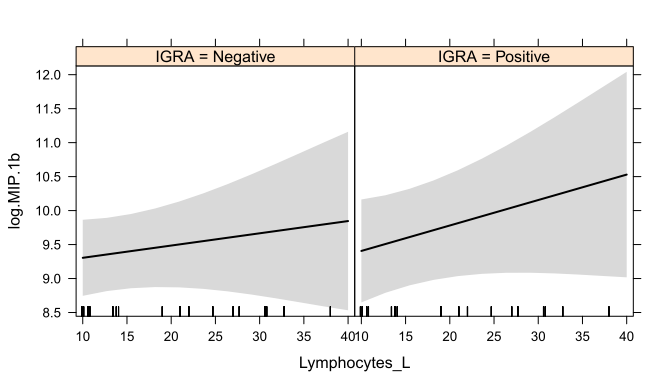 | 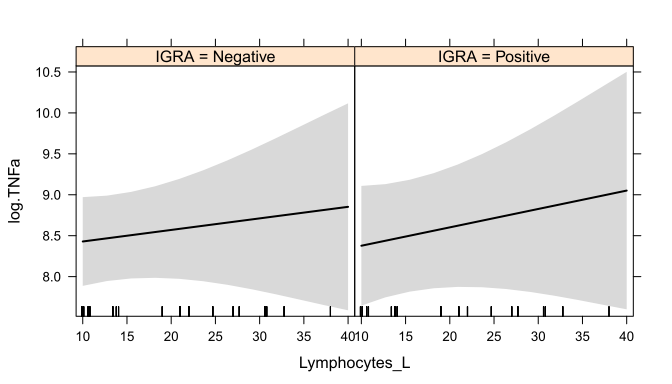 |
| 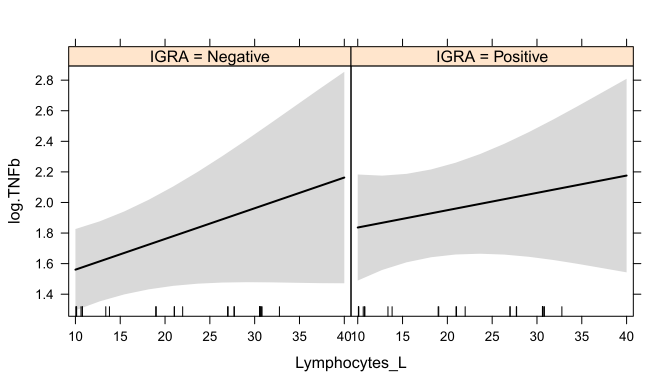 | 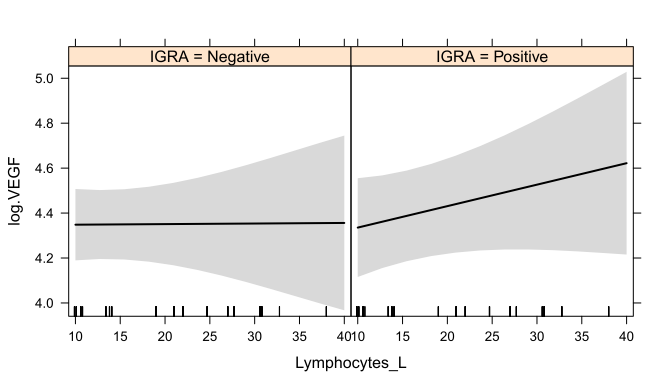 |

| **Fig S3C.** Macrophage associated cytokine concentration stratified by IGRA | |
| --- | --- |
| 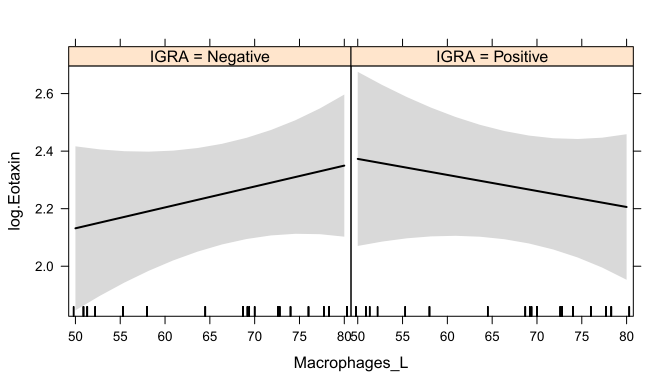 | 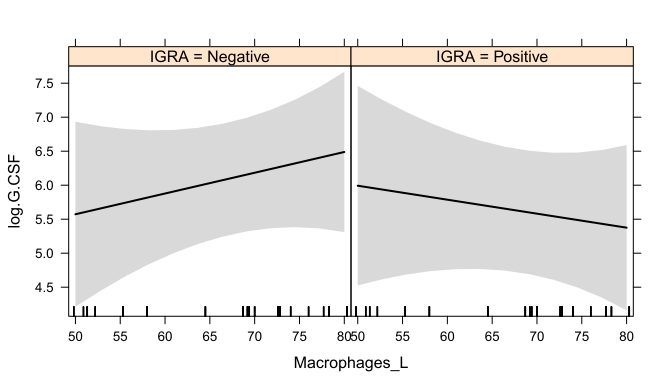 |
| 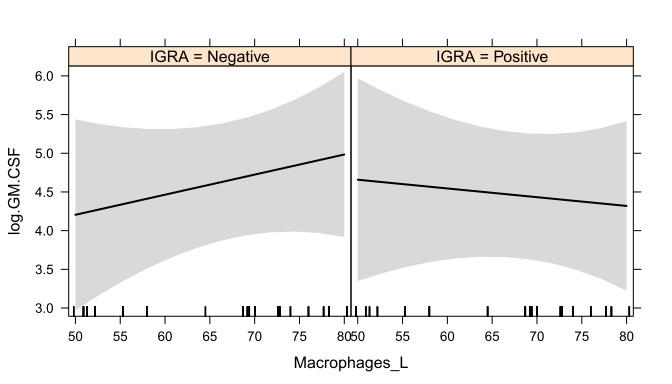 | 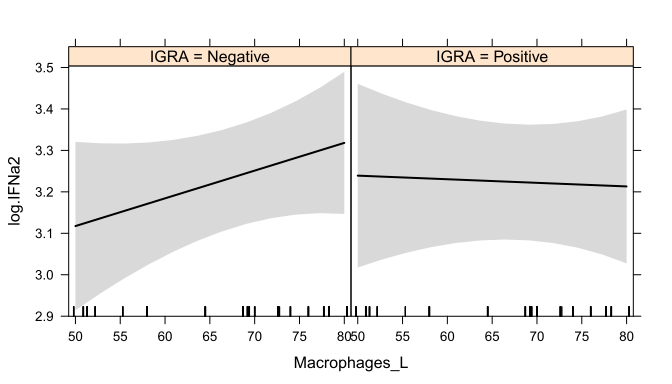 |
| 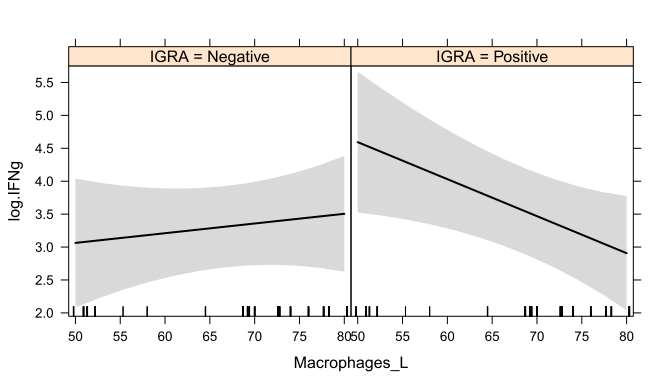 | 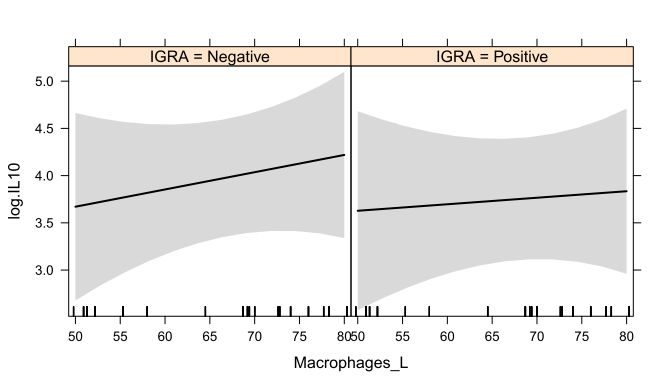 |
| 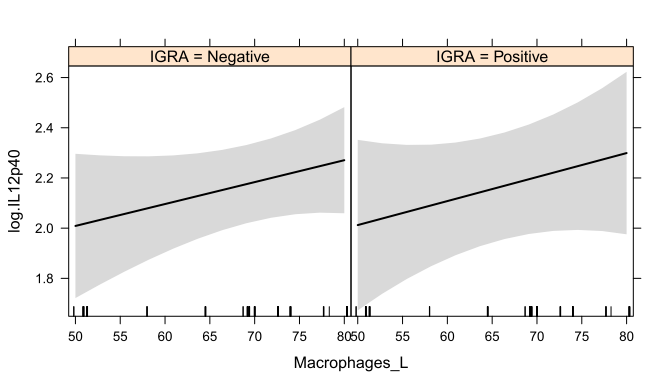 | 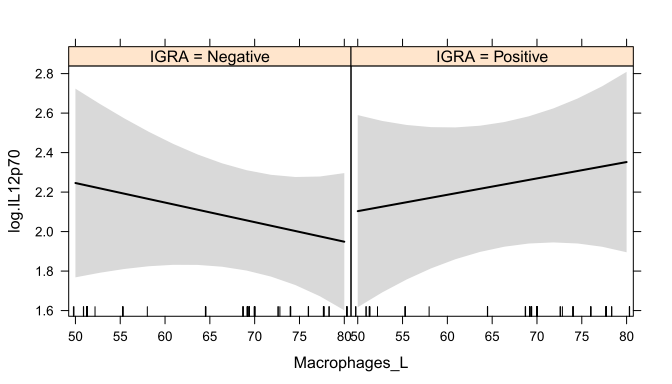 |
| 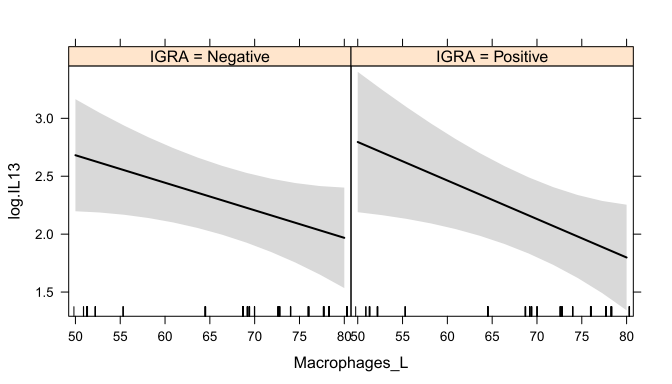 | 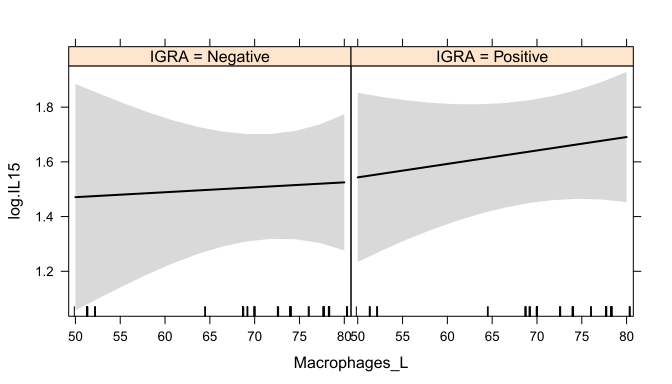 |
| 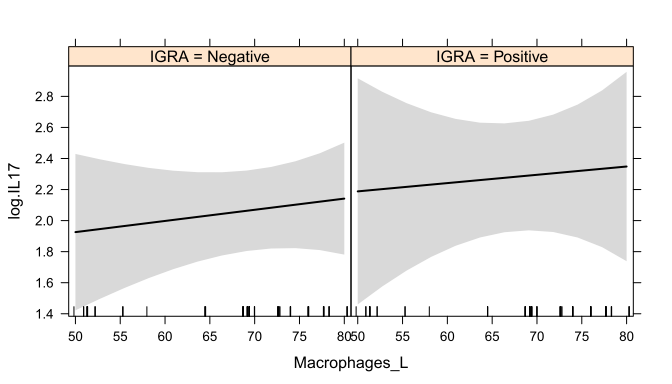 | 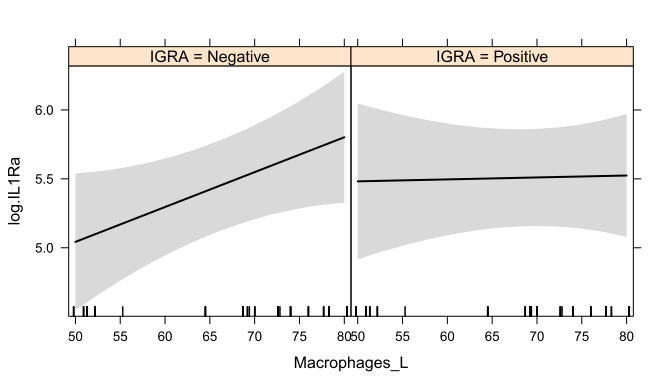 |
| 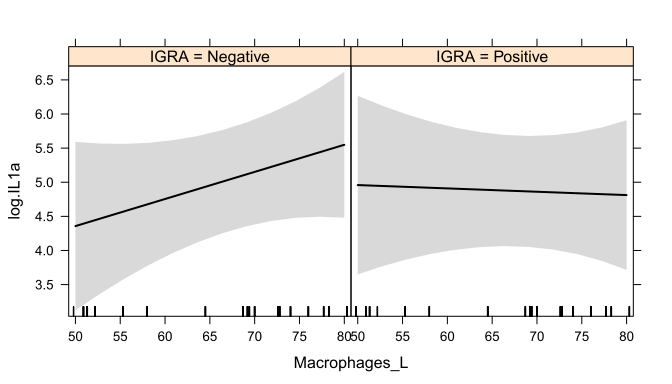 | 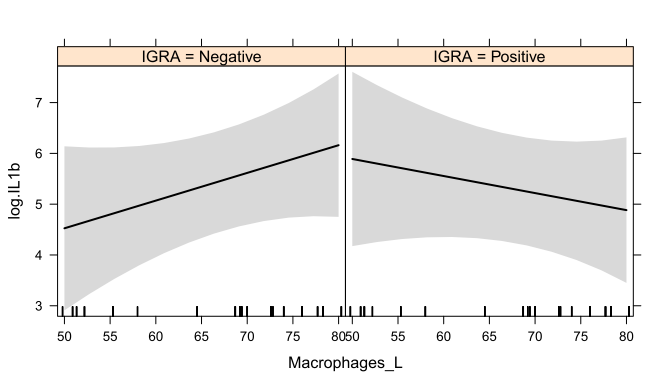 |
| 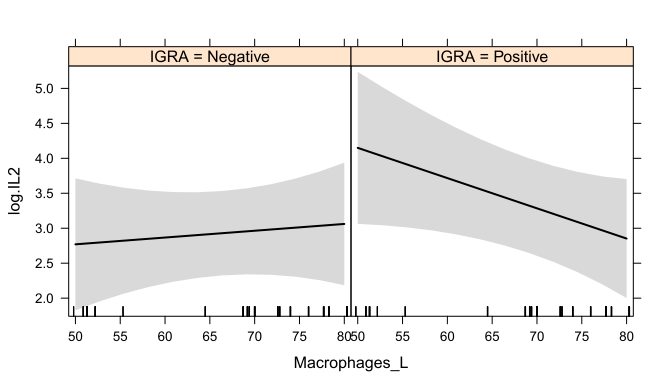 | 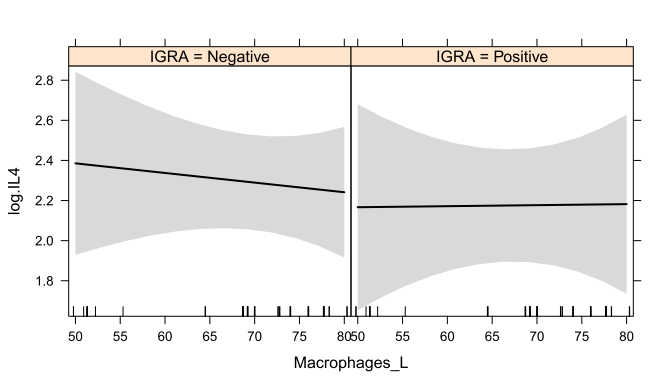 |
| 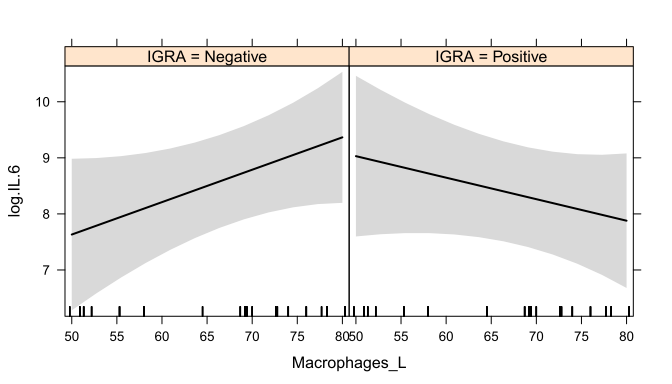 | 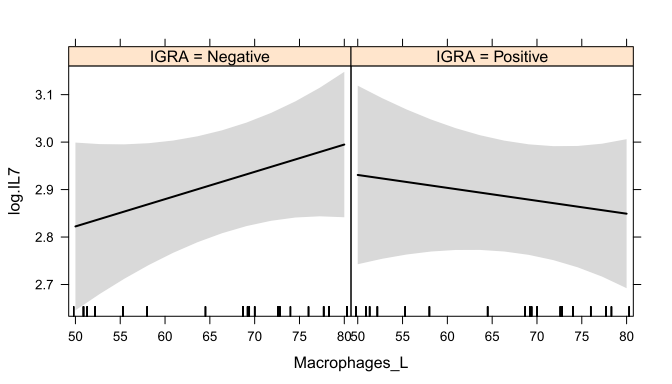 |
| 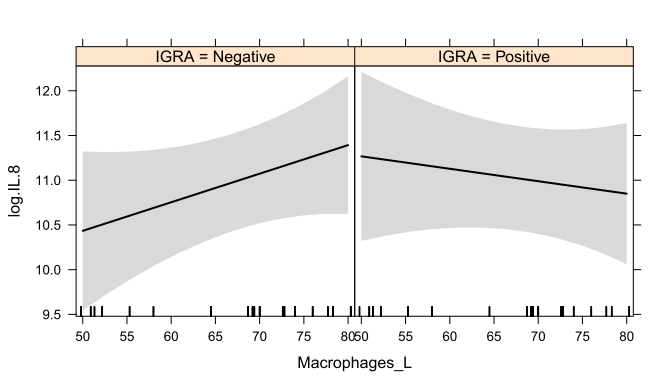 | 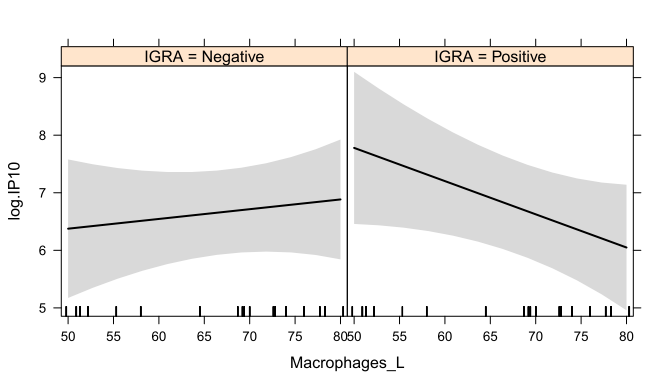 |
| 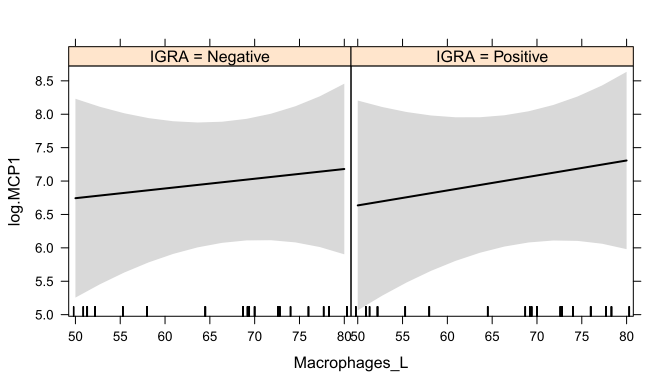 | 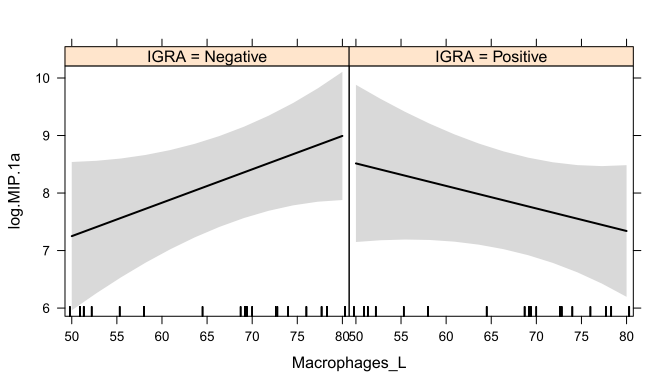 |
| 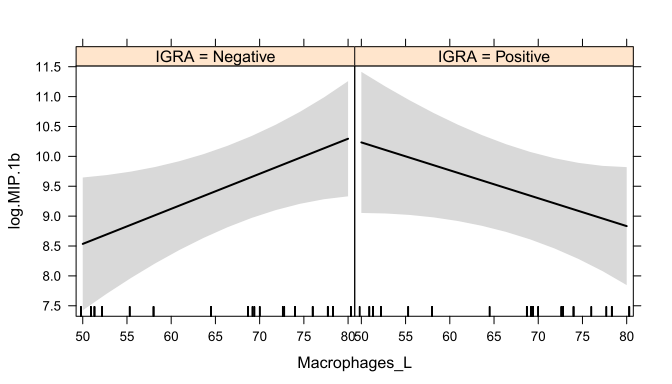 | 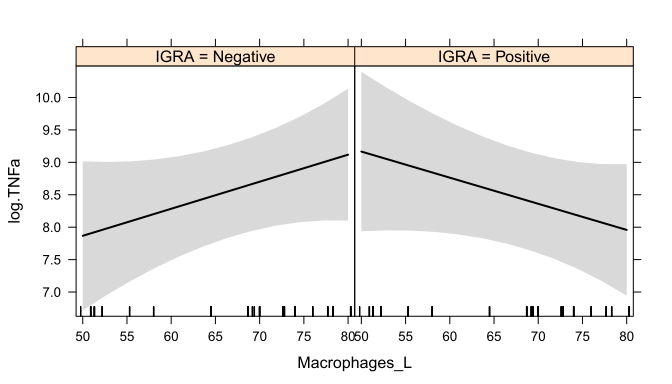 |
| 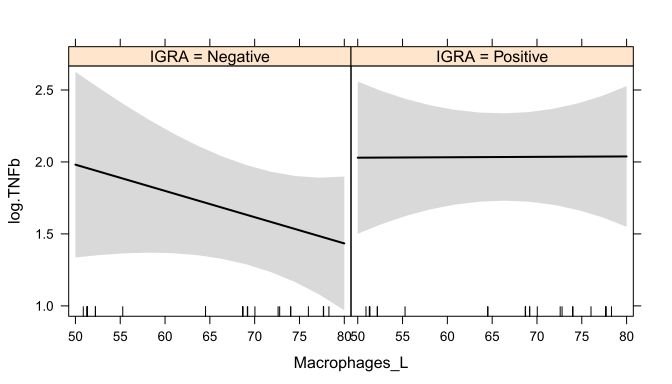 | 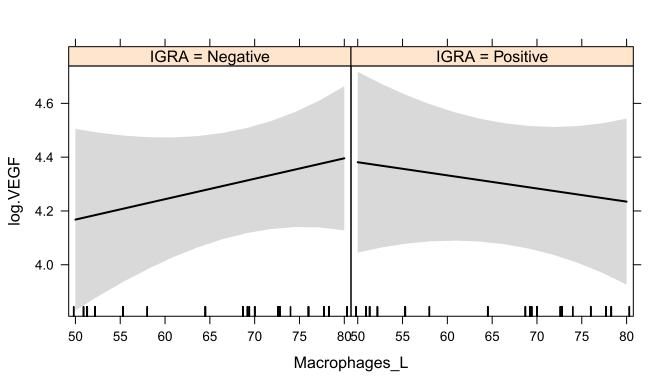 |
