# Supplementary material for: Pulmonary immune responses to Mycobacterium tuberculosis in exposed individuals
Source: PLoS One. 2017 Nov 10;12(11):e0187882. doi: 10.1371/journal.pone.0187882 (PMC5695274; doi:10.1371/journal.pone.0187882)
Supplement: S4 Fig — Estimates, with 95% confidence bands, from linear mixed-effects models of association between three cell types (A—lympocytes, B—macrophages) and cytokine concentration, stratified by BAL IGRA. (DOCX) [file pone.0187882.s005.docx]

**IGRA status and pulmonary immune responses to *Mycobacterium tuberculosis* in exposed individuals**

Christian Herzmann, Martin Ernst, Christoph Lange, Steffen Stenger, Stefan Kaufmann, Norbert Reiling, Tom Schaberg, Lize van der Merwe, Jeroen Maertzdorf for the Tb or not Tb consortium

**Supplementary figure 4**

| **Fig S4A-B.** Estimates, with 95% confidence bands , from linear mixed-effects models of association between three cell types and cytokine concentration, stratified by BAL IGRA. | |
| --- | --- |
| **Fig S4A.** Lymphocyte associated cytokine concentration stratified by BAL IGRA | |
| 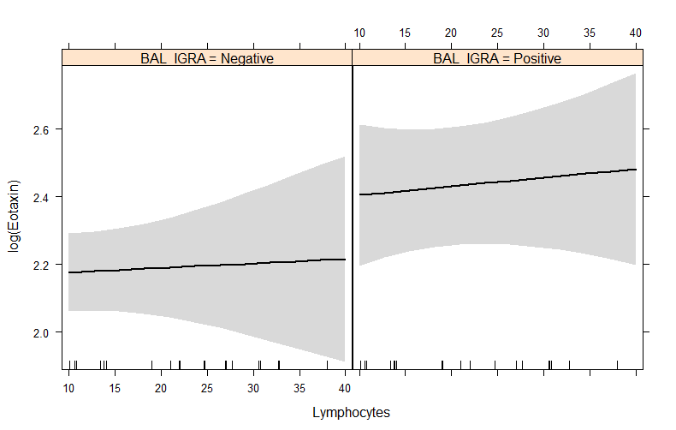 | 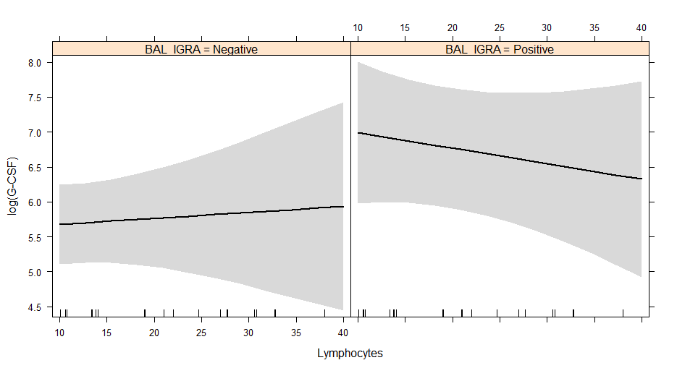 |
| 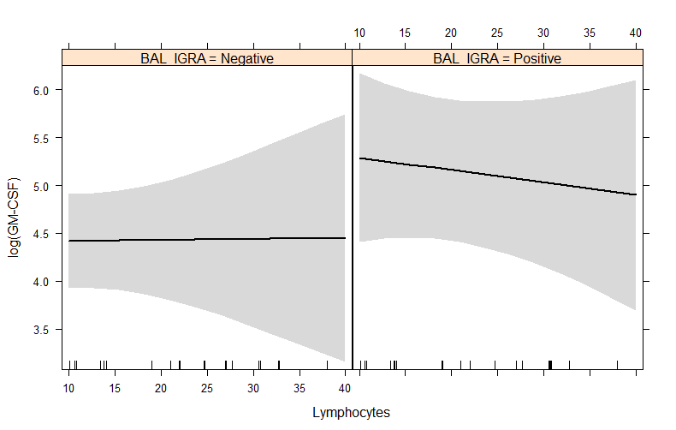 | 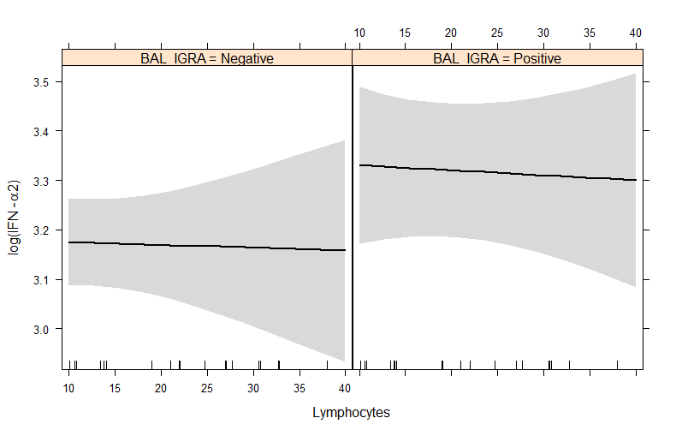 |
| 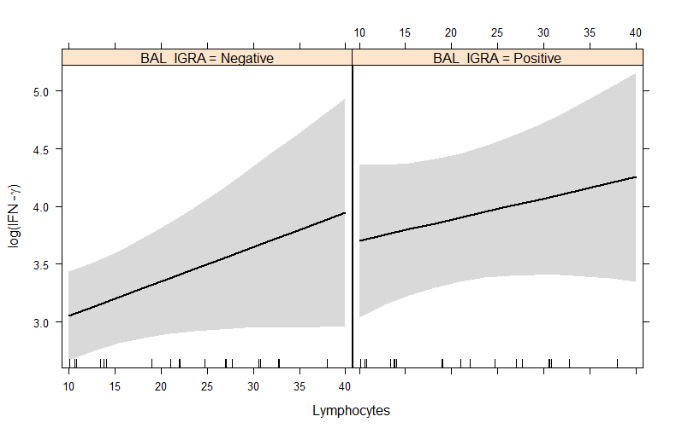 | 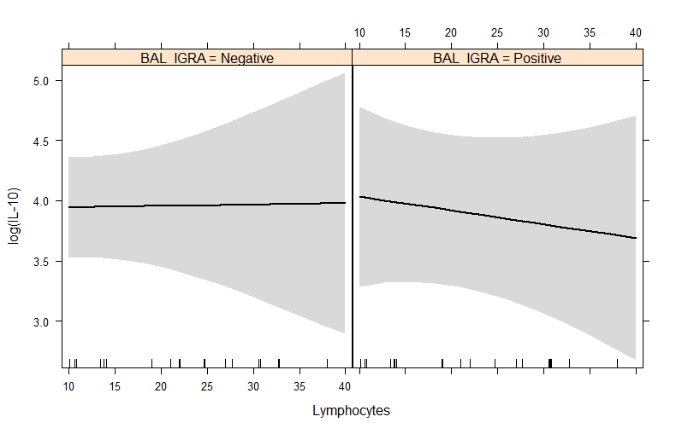 |
| 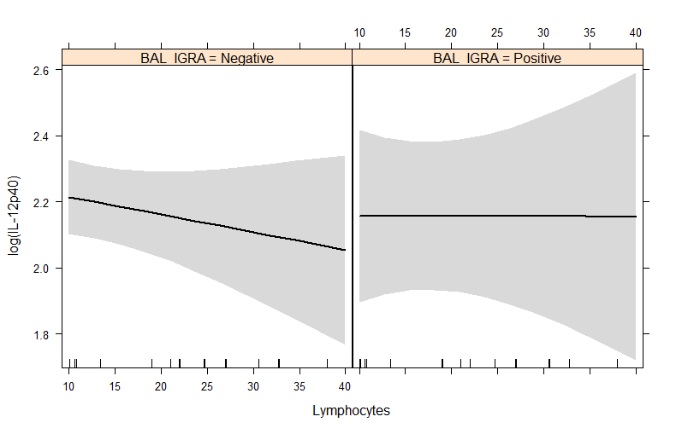 | 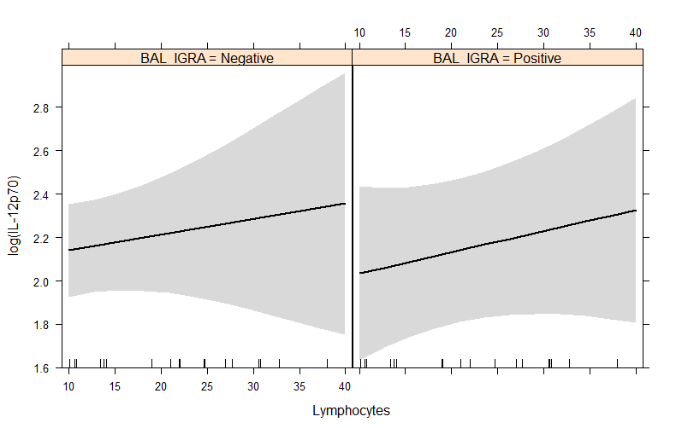 |
| 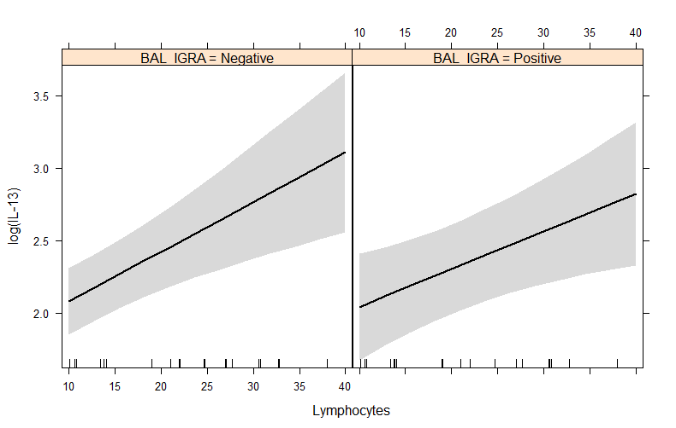 | 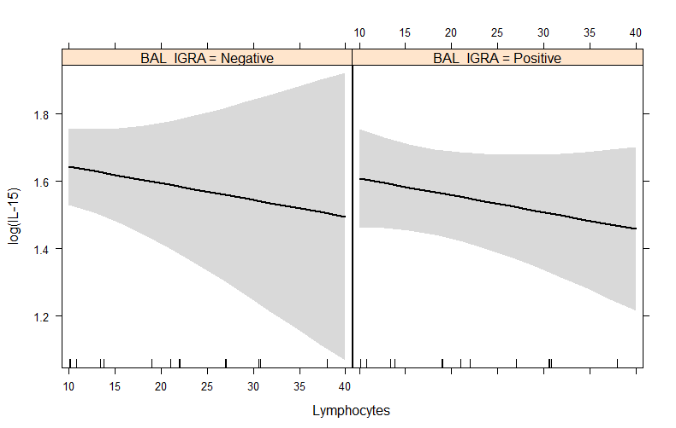 |
| 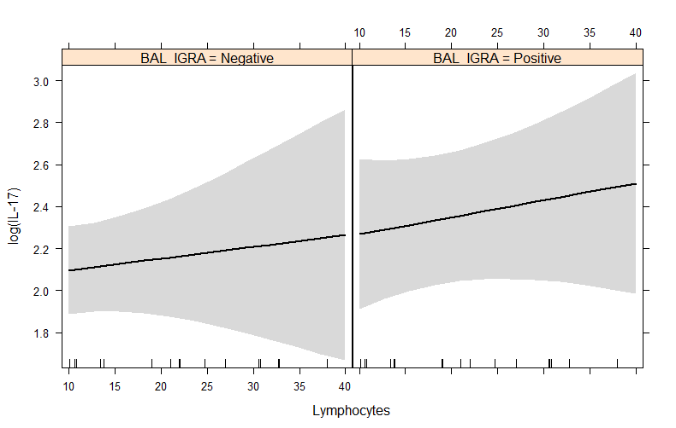 | 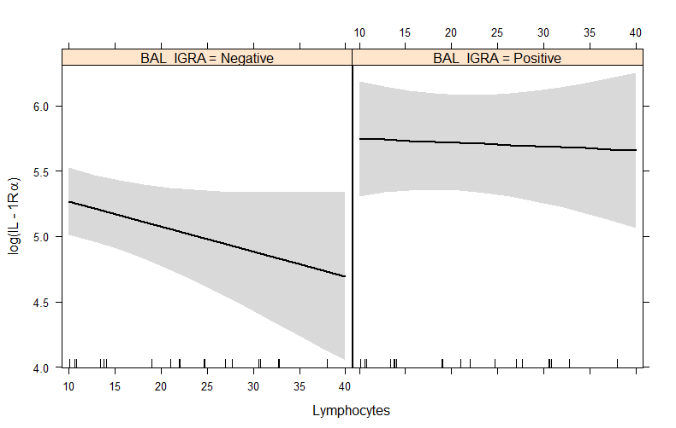 |
| 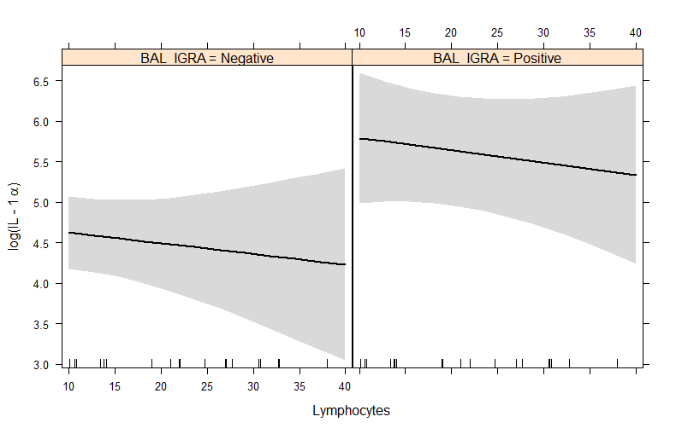 | 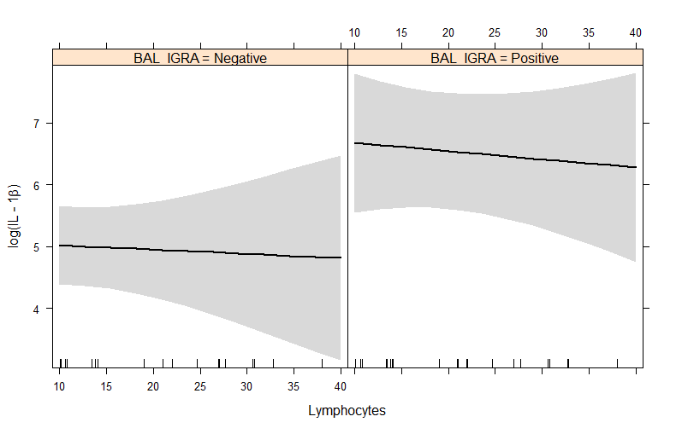 |
| 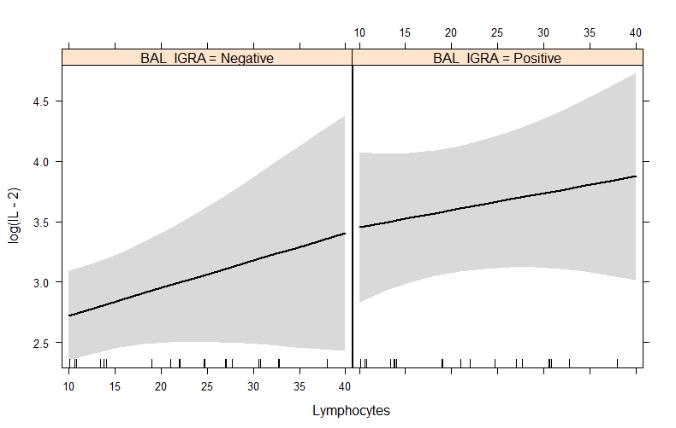 | 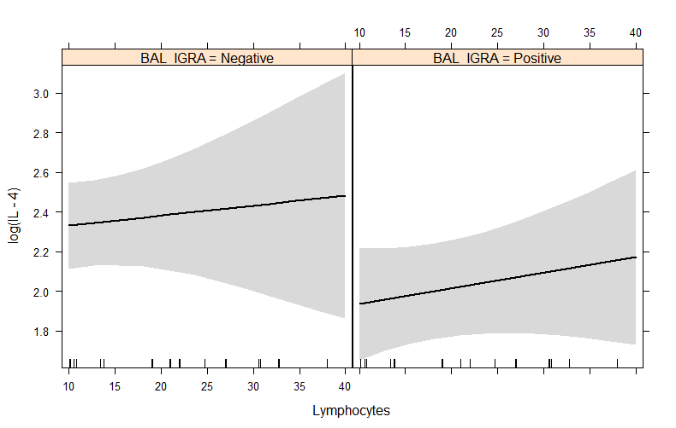 |
| 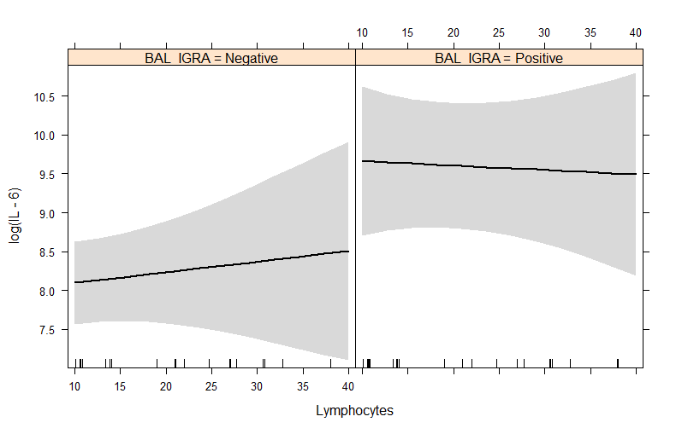 | 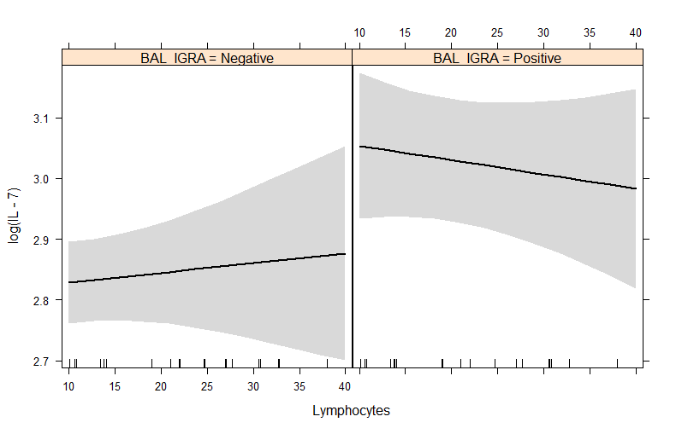 |
| 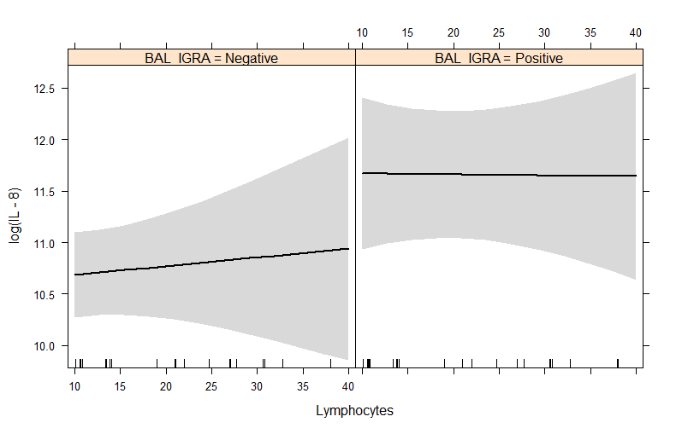 | 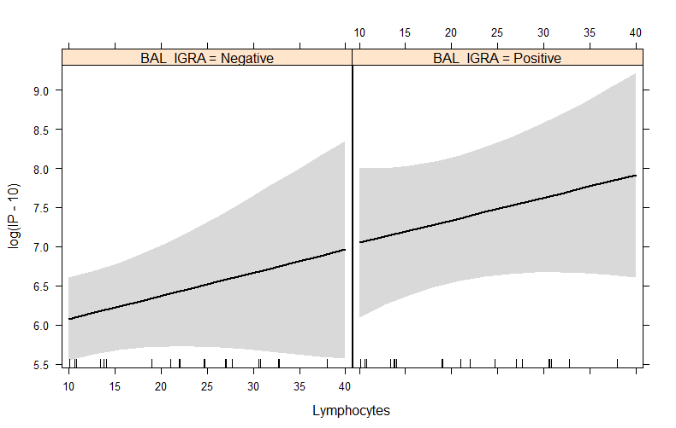 |
| 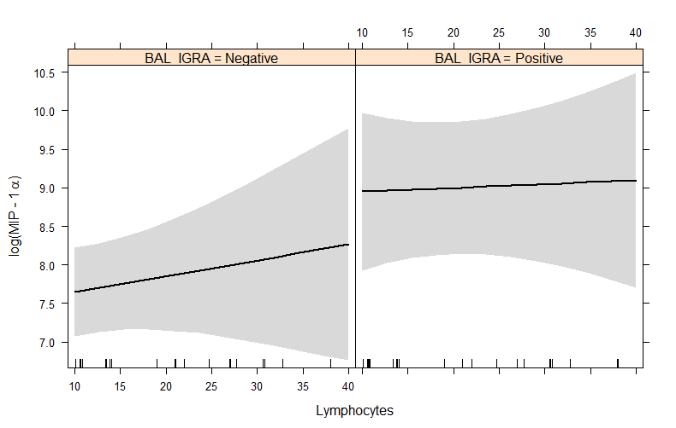 | 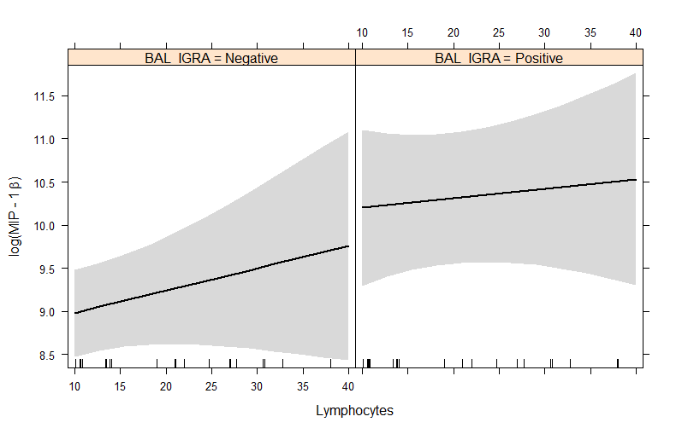 |
| 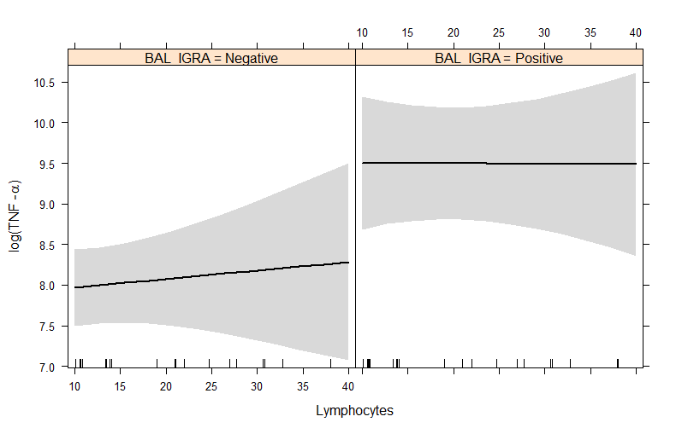 | 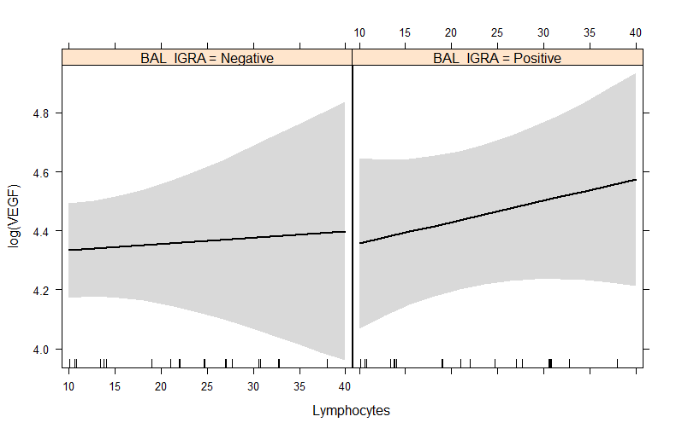 |

| **Fig S4B.** Macrophage associated cytokine concentration stratified by BAL IGRA | |
| --- | --- |
| **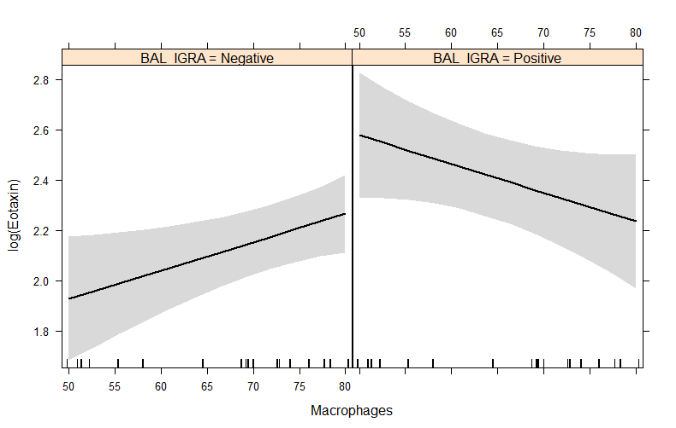** | 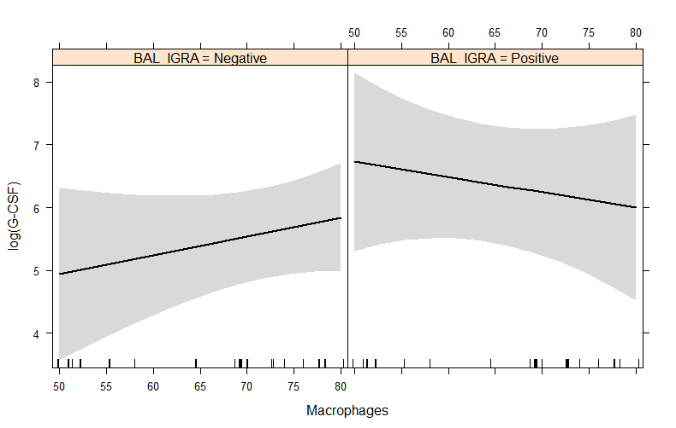 |
| **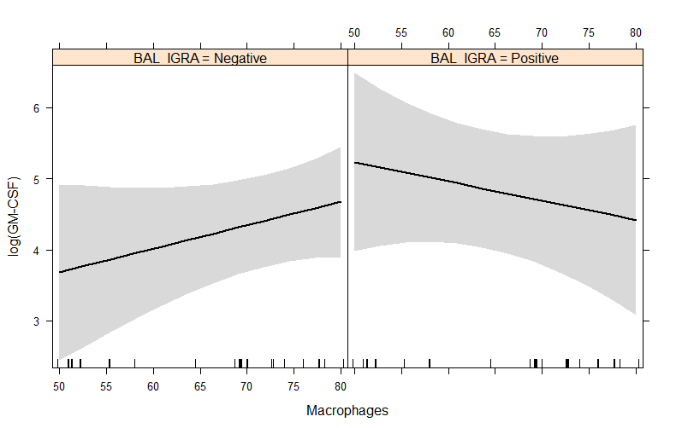** | 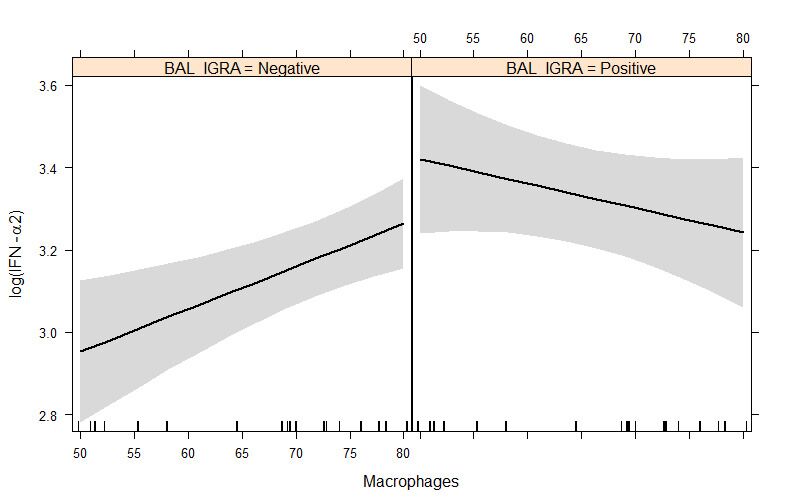 |
| **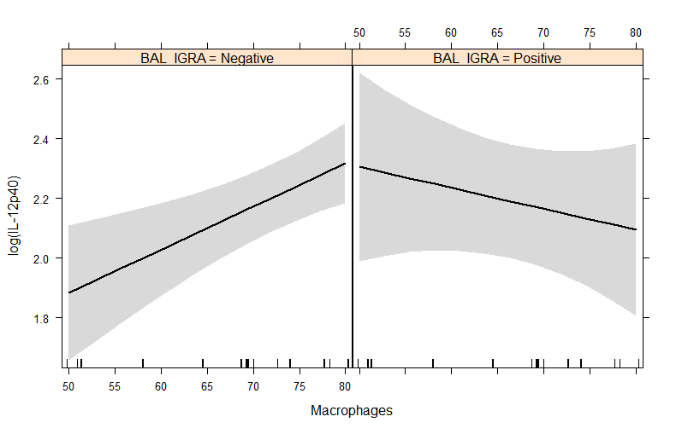** | 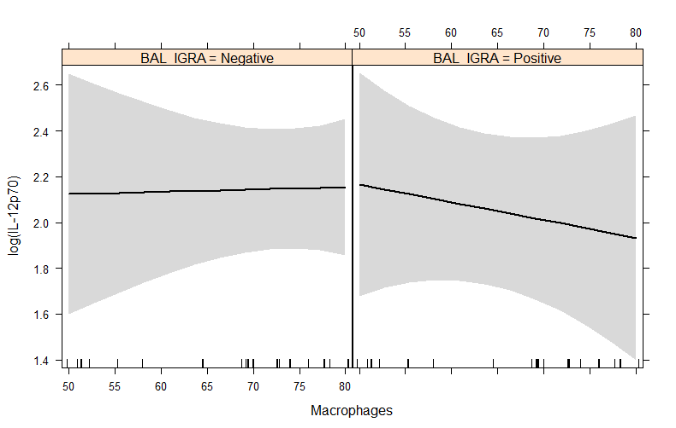 |
| **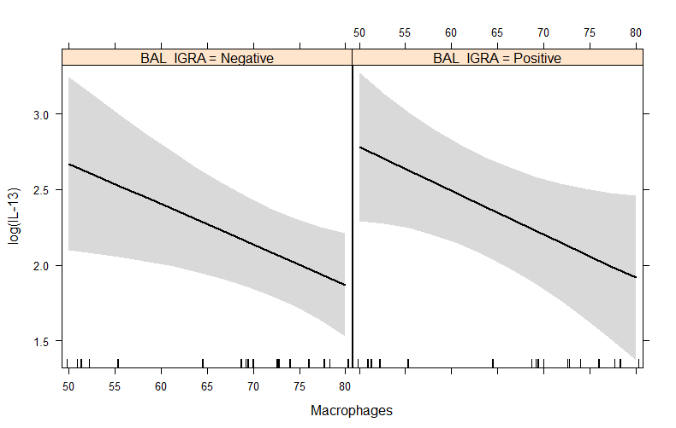** | 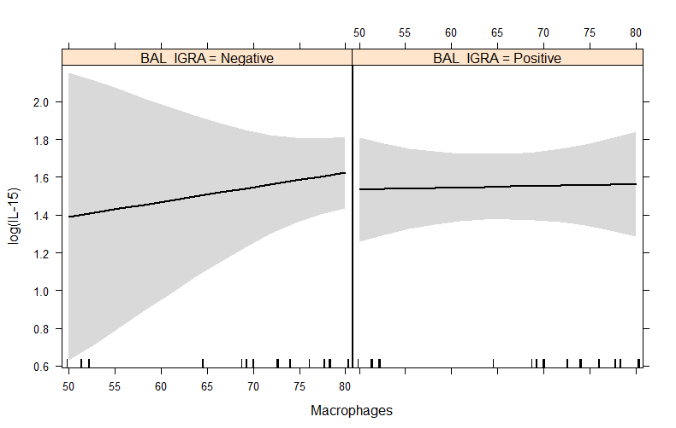 |
| **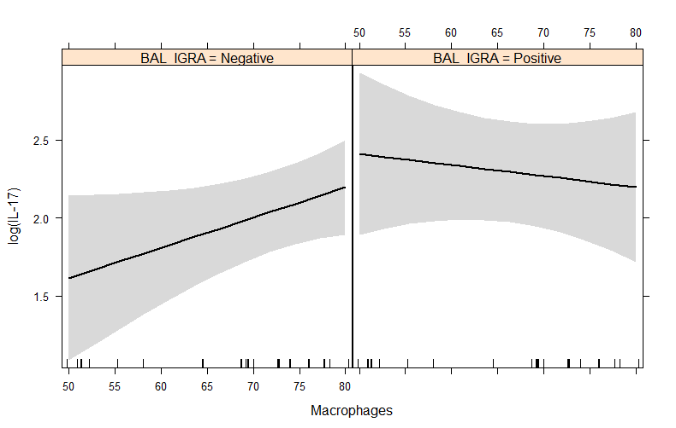** | 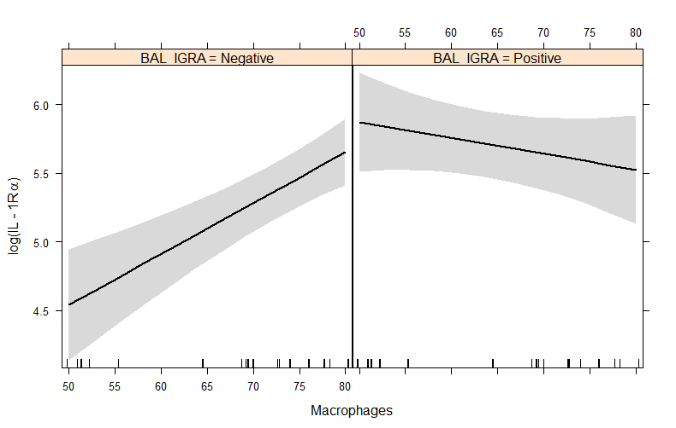 |
| **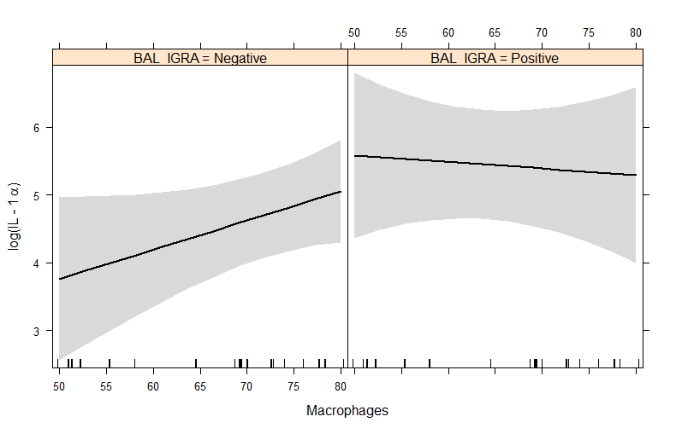** | 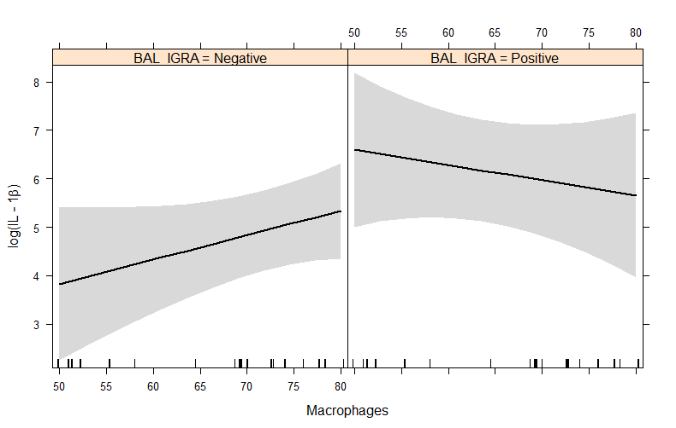 |
| **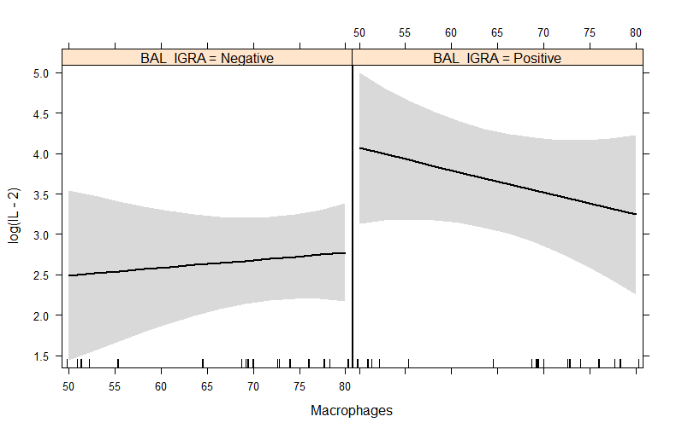** | 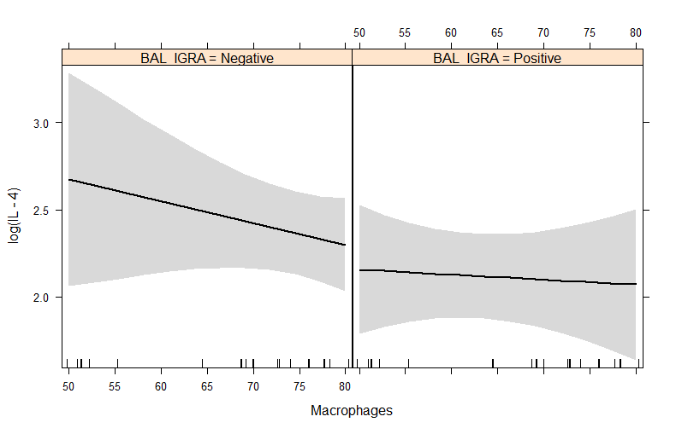 |
| **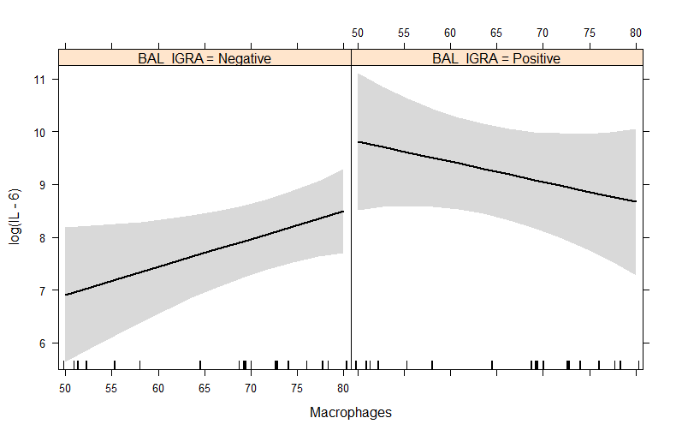** | 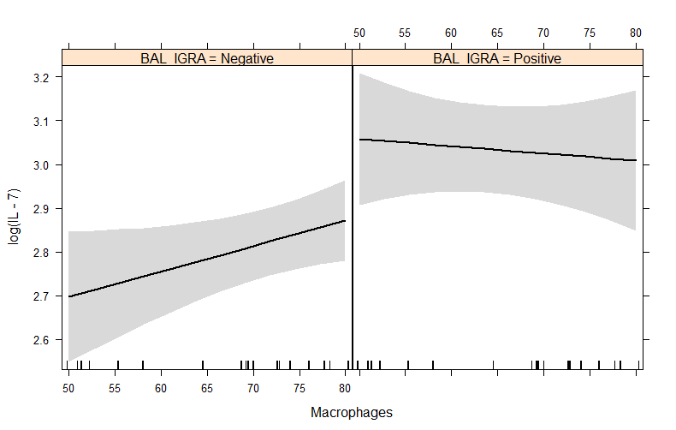 |
| **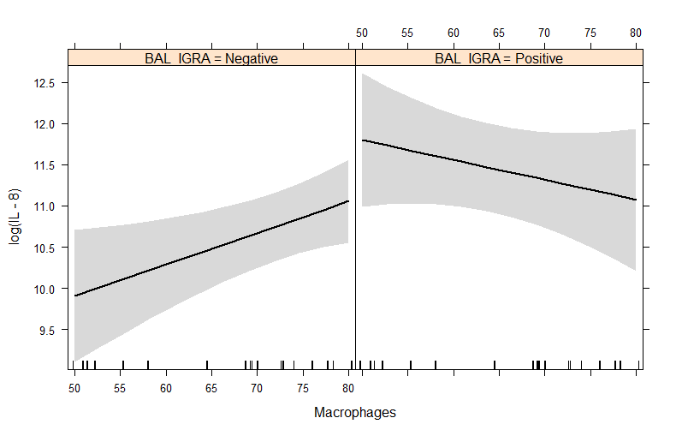** | 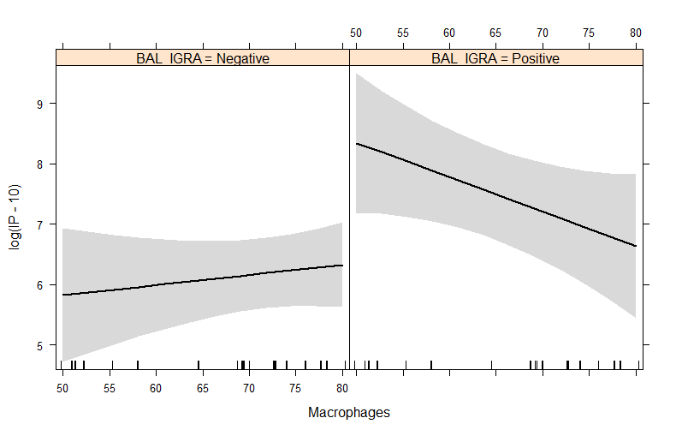 |
| **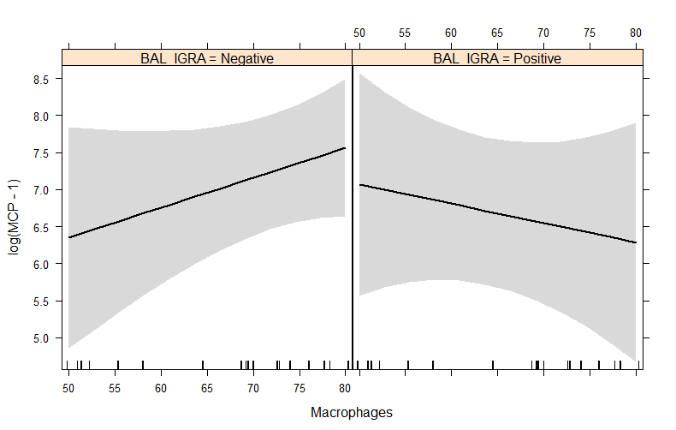** | 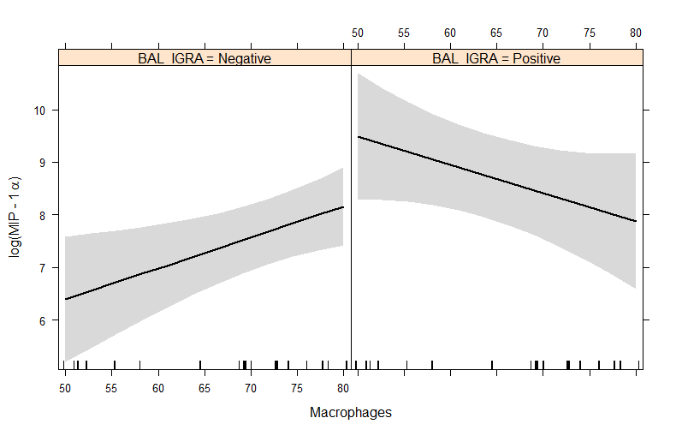 |
| **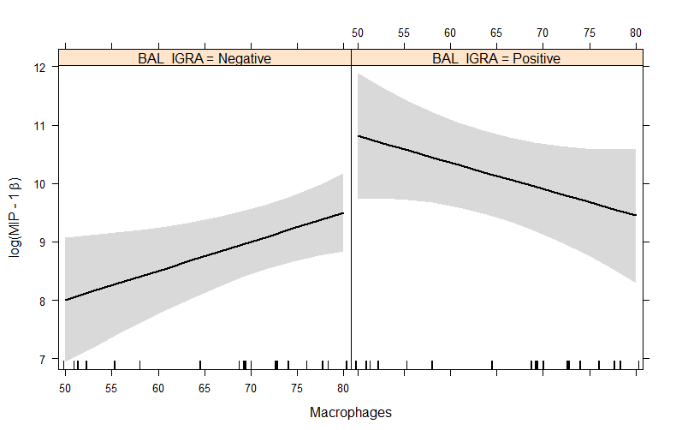** | 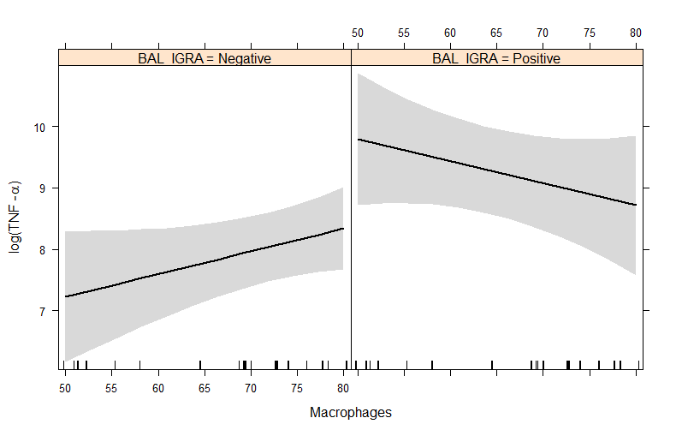 |
| **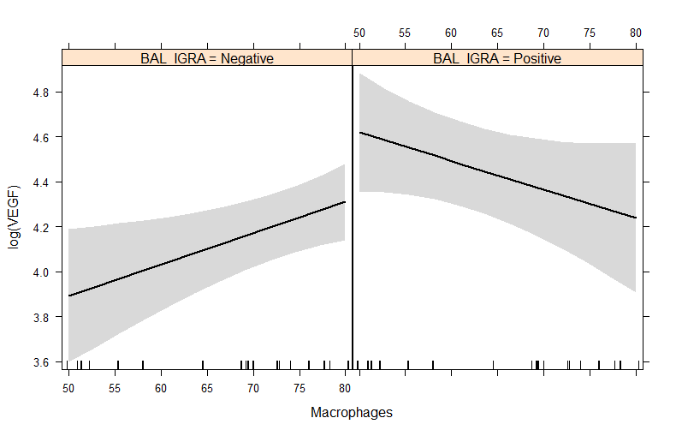** | 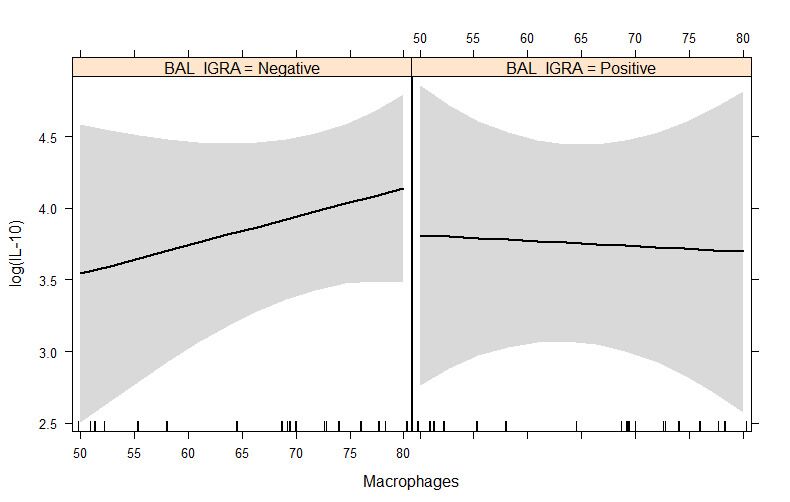 |
| 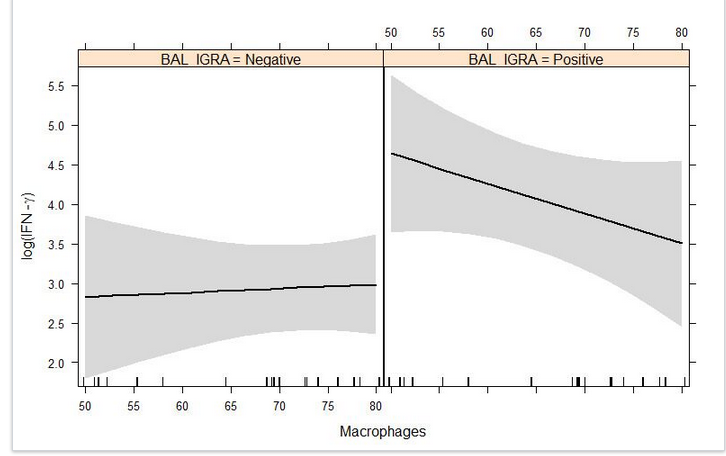 |  |
