# Supplementary material for: Pulmonary immune responses to Mycobacterium tuberculosis in exposed individuals
Source: PLoS One. 2017 Nov 10;12(11):e0187882. doi: 10.1371/journal.pone.0187882 (PMC5695274; doi:10.1371/journal.pone.0187882)
Supplement: S1 Table — (DOCX) [file pone.0187882.s006.docx]

**IGRA status and pulmonary immune responses to *Mycobacterium tuberculosis* in exposed individuals**

Christian Herzmann, Martin Ernst, Christoph Lange, Steffen Stenger, Stefan Kaufmann, Norbert Reiling, Tom Schaberg, Lize van der Merwe, Jeroen Maertzdorf for the Tb or not Tb consortium

**Supplementary table 1**

**Table S1.** P-values for changes of cytokine concentrations in BAL cell supernatants following antigen stimulation or ex-vivo infection with M. tuberculosis stains. Neither blood IGRA nor BAL IGRA status was included for this analysis.

|  | **Antigen stimulation** | | |  | **Infection** | | |  | **positive controls** | |
| --- | --- | --- | --- | --- | --- | --- | --- | --- | --- | --- |
|  | **ESAT-6** | **CFP-10** | **PPD** |  | **H37Rv** | **isol2** | **isol3** |  | **LPS** | **PHA** |
| Eotaxin | <0.0001 | <0.0001 | 0,0244 |  | 0,0001 | 0,0002 | <0.0001 |  | 0,0013 | 0,3064 |
| GCSF | 0,3365 | 0,1005 | 0,0091 |  | 0,6496 | <0.0001 | 0,0054 |  | <0.0001 | 0,0116 |
| GMCSF | 0,0202 | 0,0078 | 0,0002 |  | 0,8468 | <0.0001 | 0,0053 |  | <0.0001 | <0.0001 |
| IFNα2 | 0,1376 | 0,0501 | 0,1903 |  | 0,3882 | 0,0141 | 0,0038 |  | 0,2233 | <0.0001 |
| IFNγ | 0,0385 | 0,0800 | <0.0001 |  | <0.0001 | <0.0001 | <0.0001 |  | 0,6787 | <0.0001 |
| IL10 | 0,1148 | 0,4565 | 0,0336 |  | <0.0001 | 0,0004 | 0,1545 |  | <0.0001 | <0.0001 |
| IL12p40 | 0,1300 | 0,2477 | 0,3642 |  |  |  |  |  | 0,7620 | 0,0100 |
| IL12p70 | 0,0017 | 0,0004 | 0,0208 |  | 0,2553 | 0,4167 | 0,9268 |  | 0,0001 | 0,0261 |
| IL13 | 0,1616 | 0,3788 | 0,0656 |  | 0,9103 | 0,0059 | 0,0032 |  | 0,2236 | <0.0001 |
| IL15 | 0,3612 | 0,4553 | 0,8789 |  | 0,2588 | 0,3570 | 0,9939 |  | 0,5728 | 0,0002 |
| IL17 | 0,1482 | 0,0133 | 0,0787 |  | 0,5334 | 0,9371 | 0,3606 |  | 0,0417 | <0.0001 |
| IL1Rα | 0,8394 | 0,8621 | 0,0069 |  | <0.0001 | 0,2214 | 0,6348 |  | <0.0001 | <0.0001 |
| IL1a | 0,5002 | 0,7446 | 0,0465 |  | <0.0001 | <0.0001 | <0.0001 |  | <0.0001 | 0,0001 |
| IL1b | <0.0001 | <0.0001 | 0,0006 |  | <0.0001 | <0.0001 | <0.0001 |  | <0.0001 | 0,0001 |
| IL2 | 0,9390 | 0,4235 | <0.0001 |  | 0,0001 | <0.0001 | <0.0001 |  | 0,8004 | <0.0001 |
| IL4 | 0,9646 | 0,9217 | 0,7960 |  |  |  |  |  | 0,9896 | <0.0001 |
| IL6 | <0.0001 | <0.0001 | <0.0001 |  | 0,1311 | <0.0001 | 0,0006 |  | <0.0001 | <0.0001 |
| IL7 | 0,0355 | 0,4255 | 0,1006 |  | 0,1375 | 0,0179 | 0,0523 |  | 0,0043 | 0,3606 |
| IL8 | 0,5894 | 0,4449 | 0,0026 |  | <0.0001 | 0,4109 | 0,4850 |  | <0.0001 | 0,0073 |
| IP10 | 0,4624 | 0,3011 | <0.0001 |  | 0,0524 | <0.0001 | <0.0001 |  | 0,7163 | <0.0001 |
| MCP1 | 0,6426 | 0,3840 | <0.0001 |  | <0.0001 | 0,8960 | 0,7733 |  | 0,1718 | <0.0001 |
| MIP1α | 0,0015 | 0,0012 | 0,0005 |  | 0,6610 | 0,0001 | 0,0007 |  | <0.0001 | <0.0001 |
| MIP1b | 0,0507 | 0,0112 | <0.0001 |  | 0,0467 | 0,0036 | 0,0196 |  | <0.0001 | <0.0001 |
| TNFα | <0.0001 | <0.0001 | 0,0738 |  | 0,1326 | <0.0001 | <0.0001 |  | <0.0001 | 0,0001 |
| VEGF | <0.0001 | <0.0001 | 0,0075 |  | 0,1067 | 0,1580 | 0,3844 |  | <0.0001 | 0,0091 |
