# Supplementary material for: Pulmonary immune responses to Mycobacterium tuberculosis in exposed individuals
Source: PLoS One. 2017 Nov 10;12(11):e0187882. doi: 10.1371/journal.pone.0187882 (PMC5695274; doi:10.1371/journal.pone.0187882)
Supplement: S2 Table — (DOCX) [file pone.0187882.s007.docx]

**IGRA status and pulmonary immune responses to *Mycobacterium tuberculosis* in exposed individuals**

Christian Herzmann, Martin Ernst, Christoph Lange, Steffen Stenger, Stefan Kaufmann, Norbert Reiling, Tom Schaberg, Lize van der Merwe, Jeroen Maertzdorf for the Tb or not Tb consortium

**Supplementary table 2**

**Table S2.** P-values for cytokine concentration differences in BAL cell culture supernatants between blood IGRA positive and negative subjects

|  | **Antigen stimulation** | | |  | **Infection** | | |  | **positive controls** | |
| --- | --- | --- | --- | --- | --- | --- | --- | --- | --- | --- |
|  | **ESAT-6** | **CFP-10** | **PPD** |  | **H37Rv** | **isol2** | **isol3** |  | **LPS** | **PHA** |
| Eotaxin | 0,0390 | 0,0337 | 0,0193 |  | 0,3914 | 0,1763 | 0,8058 |  | 0,0019 | 0,0042 |
| GCSF | 0,2108 | 0,0714 | 0,5927 |  | 0,4022 | 0,7695 | 0,3792 |  | 0,9287 | 0,9789 |
| GMCSF | 0,6790 | 0,7530 | 0,2684 |  | 0,9948 | 0,9210 | 0,2727 |  | 0,1583 | 0,0793 |
| IFNα2 | 0,5280 | 0,3023 | 0,3811 |  | 0,9689 | 0,1630 | 0,8448 |  | 0,8027 | 0,7337 |
| IFNγ | 0,3875 | 0,3871 | 0,0805 |  | 0,4648 | 0,6839 | 0,7976 |  | 0,7541 | 0,7617 |
| IL10 | 0,6720 | 0,4329 | 0,2946 |  | 0,5428 | 0,7223 | 0,5835 |  | 0,0929 | 0,0709 |
| IL12p40 |  |  |  |  |  |  |  |  |  |  |
| IL12p70 | 0,2711 | 0,7339 | 0,2207 |  | 0,4149 | 0,6194 | 0,5480 |  | 0,0441 | 0,1609 |
| IL13 | 0,8961 | 0,7505 | 0,2456 |  | 0,5717 | 0,0645 | 0,2675 |  | 0,4631 | 0,7612 |
| IL15 | 0,8369 | 0,6248 | 0,2404 |  | 0,2881 | 0,1835 | 0,4540 |  | 0,0890 | 0,1472 |
| IL17 | 0,9003 | 0,4811 | 0,0165 |  | 0,8380 | 0,4330 | 0,3079 |  | 0,0545 | 0,0616 |
| IL1Rα | 0,1199 | 0,0858 | 0,0925 |  | 0,8133 | 0,1544 | 0,7947 |  | 0,2334 | 0,1486 |
| IL1a | 0,4521 | 0,3482 | 0,1081 |  | 0,1046 | 0,1942 | 0,8521 |  | 0,0329 | 0,0282 |
| IL1b | 0,6987 | 0,6468 | 0,5996 |  | 0,7736 | 0,5455 | 0,9812 |  | 0,3973 | 0,1368 |
| IL2 | 0,9664 | 0,7151 | 0,8227 |  | 0,0019 | 0,1069 | 0,0131 |  | 0,9549 | 0,6078 |
| IL4 |  |  |  |  |  |  |  |  |  |  |
| IL6 | 0,4560 | 0,4899 | 0,1673 |  | 0,7595 | 0,7203 | 0,5701 |  | 0,1192 | 0,4071 |
| IL7 | 0,0497 | 0,0315 | 0,0020 |  | 0,4949 | 0,7258 | 0,2361 |  | 0,0562 | 0,0025 |
| IL8 | 0,6198 | 0,0445 | 0,9275 |  | 0,8362 | 0,9740 | 0,9864 |  | 0,5638 | 0,6444 |
| IP10 | 0,7340 | 0,1665 | 0,5635 |  | 0,9593 | 0,7300 | 0,9571 |  | 0,7247 | 0,0895 |
| MCP1 | 0,8882 | 0,4684 | 0,8778 |  | 0,4197 | 0,2186 | 0,0974 |  | 0,7882 | 0,0670 |
| MIP1α | 0,8502 | 0,5180 | 0,4442 |  | 0,9061 | 0,6116 | 0,5998 |  | 0,4535 | 0,8931 |
| MIP1b | 0,7524 | 0,8915 | 0,6579 |  | 0,2002 | 0,4807 | 0,3036 |  | 0,1361 | 0,3728 |
| TNFα | 0,1228 | 0,0667 | 0,1549 |  | 0,8102 | 0,3841 | 0,5234 |  | 0,0816 | 0,3659 |
| VEGF | 0,7631 | 0,4413 | 0,0491 |  | 0,1496 | 0,2889 | 0,2393 |  | 0,0857 | 0,0338 |
