# Supplementary material for: Pulmonary immune responses to Mycobacterium tuberculosis in exposed individuals
Source: PLoS One. 2017 Nov 10;12(11):e0187882. doi: 10.1371/journal.pone.0187882 (PMC5695274; doi:10.1371/journal.pone.0187882)
Supplement: S3 Table — (DOCX) [file pone.0187882.s008.docx]

**IGRA status and pulmonary immune responses to *Mycobacterium tuberculosis* in exposed individuals**

Christian Herzmann, Martin Ernst, Christoph Lange, Steffen Stenger, Stefan Kaufmann, Norbert Reiling, Tom Schaberg, Lize van der Merwe, Jeroen Maertzdorf for the Tb or not Tb consortium

**Supplementary table 3**

**Table S3.** P-values for cytokine concentration differences in BAL cell culture supernatants between BAL IGRA positive and negative subjects

|  | **Antigen stimulation** | | |  | **Infection** | | |  | **positive controls** | |
| --- | --- | --- | --- | --- | --- | --- | --- | --- | --- | --- |
|  | **ESAT-6** | **CFP-10** | **PPD** |  | **H37Rv** | **isol2** | **isol3** |  | **LPS** | **PHA** |
| Eotaxin | 0,2275 | 0,2491 | 0,2733 |  | 0,3440 | 0,8193 | 0,1114 |  | 0,9774 | 0,3387 |
| GCSF | 0,8835 | 0,6201 | 0,9305 |  | 0,3692 | 0,2416 | 0,6612 |  | 0,1056 | 0,9985 |
| GMCSF | 0,9368 | 0,9316 | 0,7301 |  | 0,3085 | 0,2818 | 0,8400 |  | 0,3796 | 0,7727 |
| IFNα2 | 0,7062 | 0,4310 | 0,5357 |  | 0,0082 | 0,0700 | 0,6059 |  | 0,1431 | 0,8109 |
| IFNγ | 0,5283 | 0,5549 | 0,0063 |  | 0,0962 | 0,0446 | 0,0011 |  | 0,6967 | 0,1715 |
| IL10 | 0,7141 | 0,5915 | 0,4722 |  | 0,2009 | 0,3761 | 0,8656 |  | 0,4452 | 0,1081 |
| IL12p40 | 0,2288 | 0,8053 | 0,2943 |  | 0,2584 | 0,0207 | 0,9573 |  | 0,3538 | 0,5675 |
| IL12p70 | 0,0074 | 0,0849 | 0,9112 |  | 0,6016 | 0,0705 | 0,3979 |  | 0,8256 | 0,4441 |
| IL13 | 0,8969 | 0,9723 | 0,1888 |  | 0,2065 | 0,0308 | 0,0008 |  | 0,3807 | 0,0859 |
| IL15 | 0,3014 | 0,9115 | 0,3344 |  |  | 0,1926 | 0,6853 |  | 0,6851 | 0,7590 |
| IL17 |  | 0,1120 | 0,6419 |  | 0,2471 | 0,9024 | 0,2186 |  | 0,5576 | 0,1885 |
| IL1Rα | 0,5004 | 0,6827 | 0,9198 |  | 0,1342 | 0,1834 | 0,5519 |  | 0,0817 | 0,9803 |
| IL1a | 0,3174 | 0,3823 | 0,5447 |  | 0,1638 | 0,3408 | 0,9930 |  | 0,1102 | 0,7527 |
| IL1b | 0,5835 | 0,7604 | 0,4876 |  | 0,3936 | 0,4398 | 0,8338 |  | 0,3217 | 0,9754 |
| IL2 | 0,3402 | 0,2307 | 0,0217 |  | <0.0001 | <0.0001 | <0.0001 |  | 0,7846 | 0,0351 |
| IL4 | 0,6398 | 0,6618 | 0,5745 |  | 0,9408 | 0,5301 | 0,4824 |  | 0,6595 | 0,6598 |
| IL6 | 0,6837 | 0,6561 | 0,4077 |  | 0,1189 | 0,2517 | 0,9605 |  | 0,0020 | 0,0959 |
| IL7 | 0,6135 | 0,3585 | 0,8208 |  | 0,3283 | 0,2214 | 0,9954 |  | 0,0818 | 0,8517 |
| IL8 | 0,3932 | 0,4428 | 0,0771 |  | 0,6331 | 0,7547 | 0,1453 |  | 0,0152 | 0,0343 |
| IP10 | 0,9697 | 0,3420 | 0,4814 |  | 0,1291 | 0,6154 | 0,8787 |  | 0,3958 | 0,0650 |
| MCP1 | 0,6232 | 0,4683 | 0,9382 |  | 0,0014 | 0,9365 | 0,4659 |  | 0,0317 | 0,8074 |
| MIP1α | 0,9645 | 0,9376 | 0,5209 |  | 0,0057 | 0,1636 | 0,9307 |  | 0,0152 | 0,2043 |
| MIP1b | 0,4502 | 0,5558 | 0,3681 |  | 0,0267 | 0,2688 | 0,9906 |  | 0,0213 | 0,0243 |
| TNFα | 0,5074 | 0,9355 | 0,4909 |  | 0,0523 | 0,1916 | 0,7131 |  | 0,0995 | 0,9491 |
| VEGF | 0,9928 | 0,4183 | 0,0680 |  | 0,7289 | 0,2890 | 0,0552 |  | 0,8794 | 0,2170 |
