# Supplementary material for: Pulmonary immune responses to Mycobacterium tuberculosis in exposed individuals
Source: PLoS One. 2017 Nov 10;12(11):e0187882. doi: 10.1371/journal.pone.0187882 (PMC5695274; doi:10.1371/journal.pone.0187882)
Supplement: S4 Table — (DOCX) [file pone.0187882.s009.docx]

**IGRA status and pulmonary immune responses to *Mycobacterium tuberculosis* in exposed individuals**

Christian Herzmann, Martin Ernst, Christoph Lange, Steffen Stenger, Stefan Kaufmann, Norbert Reiling, Tom Schaberg, Lize van der Merwe, Jeroen Maertzdorf for the Tb or not Tb consortium

**Supplementary table 4**

**Table S4.** P-values for cytokine concentration differences in unstimulated versus infected BAL cell culture supernatants at baseline

|  |  | **Blood IGRA** | |  | **BAL IGRA** | |
| --- | --- | --- | --- | --- | --- | --- |
|  |  | **unstim.** | **uninf.** |  | **unstim.** | **uninf.** |
| Eotaxin |  | 0,0901 | 0,4221 |  | 0,2720 | 0,0258 |
| GCSF |  | 0,9055 | 0,2215 |  | 0,0144 | 0,0766 |
| GMCSF |  | 0,4353 | 0,4948 |  | 0,0448 | 0,1322 |
| IFNα2 |  | 0,6687 | 0,4462 |  | 0,2192 | 0,0360 |
| IFNγ |  | 0,7643 | 0,5021 |  | 0,3832 | 0,5943 |
| IL-10 |  | 0,2715 | 0,6393 |  | 0,4578 | 0,8401 |
| IL-12p40 |  | N/A | N/A |  | 0,6178 | 0,9438 |
| IL-12p70 |  | 0,8436 | 0,4270 |  | 0,6664 | 0,2772 |
| IL-13 |  | 0,8006 | 0,6758 |  | 0,9836 | 0,9788 |
| IL-15 |  | 0,4459 | 0,5695 |  | 0,6568 | 0,7577 |
| IL-17 |  | 0,5285 | 0,2950 |  | 0,5343 | 0,2880 |
| IL-1Rα |  | 0,9545 | 0,5177 |  | 0,1383 | 0,4464 |
| IL-1α |  | 0,4503 | 0,4016 |  | 0,0593 | 0,0021 |
| IL-1ẞ |  | 0,8296 | 0,7441 |  | 0,0084 | 0,0249 |
| IL-2 |  | 0,2124 | 0,7418 |  | 0,7927 | 0,2748 |
| IL-4 |  | N/A | N/A |  | 0,9507 | 0,6123 |
| IL-6 |  | 0,5224 | 0,7511 |  | 0,0004 | 0,0181 |
| IL-7 |  | 0,0156 | 0,9869 |  | 0,0500 | 0,0082 |
| IL-8 |  | 0,3482 | 0,7701 |  | 0,0008 | 0,4992 |
| IP10 |  | 0,4968 | 0,7495 |  | 0,0373 | 0,1024 |
| MCP1 |  | 0,3562 | 0,2858 |  | 0,7822 | 0,8462 |
| MIP1α |  | 0,8767 | 0,7200 |  | 0,0006 | 0,0743 |
| MIP1ẞ |  | 0,9970 | 0,7686 |  | 0,0002 | 0,0689 |
| TNFα |  | 0,5815 | 0,4861 |  | 0,0031 | 0,0092 |
| VEGF |  | 0,1934 | 0,8653 |  | 0,9995 | 0,6586 |
